# Lutetium background radiation in total-body PET—A simulation study on opportunities and challenges in PET attenuation correction

**DOI:** 10.3389/fnume.2022.963067

**Published:** 2022-08-10

**Authors:** Negar Omidvari, Li Cheng, Edwin K. Leung, Yasser G. Abdelhafez, Ramsey D. Badawi, Tianyu Ma, Jinyi Qi, Simon R. Cherry

**Affiliations:** ^1^Department of Biomedical Engineering, University of California, Davis, Davis, CA, United States; ^2^Department of Engineering Physics, Tsinghua University, Beijing, China; ^3^Department of Radiology, University of California, Davis, Davis, CA, United States; ^4^United Imaging Healthcare America Inc., Houston, TX, United States; ^5^Nuclear Medicine Unit, South Egypt Cancer Institute, Assiut University, Asyut, Egypt

**Keywords:** positron emission tomography (PET), total-body PET, attenuation correction, lutetium background, iterative image reconstruction

## Abstract

The current generation of total-body positron emission tomography (PET) scanners offer significant sensitivity increase with an extended axial imaging extent. With the large volume of lutetium-based scintillation crystals that are used as detector elements in these scanners, there is an increased flux of background radiation originating from ^176^Lu decay in the crystals and higher sensitivity for detecting it. Combined with the ability of scanning the entire body in a single bed position, this allows more effective utilization of the lutetium background as a transmission source for estimating 511 keV attenuation coefficients. In this study, utilization of the lutetium background radiation for attenuation correction in total-body PET was studied using Monte Carlo simulations of a 3D whole-body XCAT phantom in the uEXPLORER PET scanner, with particular focus on ultralow-dose PET scans that are now made possible with these scanners. Effects of an increased acceptance angle, reduced scan durations, and Compton scattering on PET quantification were studied. Furthermore, quantification accuracy of lutetium-based attenuation correction was compared for a 20-min scan of the whole body on the uEXPLORER, a one-meter-long, and a conventional 24-cm-long scanner. Quantification and lesion contrast were minimally affected in both long axial field-of-view scanners and in a whole-body 20-min scan, the mean bias in all analyzed organs of interest were within a ±10% range compared to ground-truth activity maps. Quantification was affected in certain organs, when scan duration was reduced to 5 min or a reduced acceptance angle of 17° was used. Analysis of the Compton scattered events suggests that implementing a scatter correction method for the transmission data will be required, and increasing the energy threshold from 250 keV to 290 keV can reduce the computational costs and data rates, with negligible effects on PET quantification. Finally, the current results can serve as groundwork for transferring lutetium-based attenuation correction into research and clinical practice.

## Introduction

Positron emission tomography (PET) is a powerful molecular imaging technique in nuclear medicine, which is widely used as a clinical and research tool for diagnosis, prognosis, and treatment planning in oncology (1, 2), with additional applications including metabolic, neurologic, cardiovascular, atherosclerosis, musculoskeletal, and infectious disease imaging (1–8). Two main constraining factors in utilizing PET broadly have been the low sensitivity in conventional PET scanners and concerns about the radiation dose. Although PET offers high sensitivity among molecular imaging techniques, only a small fraction (< 1%) of emitted 511 keV coincident photon pairs are detected in conventional clinical PET scanners (3), as with an axial-field-of-view (AFOV) of 15–30 cm they can only collect signal from a small portion of the body at a given bed position. Among recently developed long-AFOV PET scanners (9–12), the uEXPLORER is one of the two commercialized systems and the world's first total-body PET scanner that can simultaneously image the entire body (13). With a 194-cm AFOV, the uEXPLORER offers 15–68-fold increase in effective sensitivity compared to conventional short-AFOV scanners, enabling use of new scan protocols with ultrashort time frames or ultralow-dose scans, as well as unprecedented image quality with standard scan protocols (3, 7, 11, 13, 14).

Among many data corrections that are applied to PET raw data, attenuation correction (AC) can be considered as the most essential step, with the largest impact on the subsequent corrections and ultimate quantification. The fundamental step is to generate accurate attenuation maps (μ-maps)—containing the linear attenuation coefficient estimates of the object at a photon energy of 511 keV—that are used both for PET AC and scatter correction (SC). The gold standard for measuring the linear attenuation coefficients of the object at 511 keV is to use an external positron-emitting transmission source, which, in practice, requires lengthy scans, and, like any other sequential scan, it suffers from spatial mismatch due to subject motion. In the 1990s, combining PET scanners with a computed tomography (CT) scanner was proposed and was soon widely adopted (15). In PET/CT scanners, μ-maps are derived from the CT scan performed prior to the PET scan. Although CT scans are short and CT images often have diagnostic value, they come with additional radiation dose, and AC artifacts are often observed due to subject motion occurring after the CT acquisition. Furthermore, since polychromatic x-rays of energy not more than 140 keV are used in CT, the images need to be scaled to estimate the μ-map at 511 keV (16, 17). In many research studies, which may involve PET/CT scans at multiple time points, the CT images are solely used to provide the photon μ-maps essential for PET AC and SC. Therefore, there has been interest in methods that measure the attenuation coefficients simultaneously with PET acquisition without incurring additional radiation dose (18). This becomes of particular interest for ultralow-dose PET/CT scans, now made possible for the first time with the new generation of total-body PET scanners, in which the radiation dose from CT can be dominant (3).

Among these alternative methods for generating the μ-maps, emission-based, deep learning-based, and lutetium background-based techniques have shown promising results. Many emission-based techniques, such as the maximum likelihood reconstruction of attenuation and activity (MLAA) algorithm (18–20), use maximum likelihood approaches, in which the μ-map is first updated by a maximum likelihood for transmission tomography (MLTR) algorithm (21) and, subsequently, the activity map is updated by maximum likelihood expectation maximization (MLEM) (22) or ordered subset expectation maximization (OSEM) (23). Besides the computational cost, these approaches require some *a priori* knowledge about the μ-map, and presence of local maxima in the likelihood function can result in crosstalk artifacts, in which errors in the activity image are associated with errors in the μ-map. Although incorporating time-of-flight (TOF) into MLAA theoretically eliminates the crosstalk problem, it does not eliminate the possibility of other local maxima (19), and it only determines the μ-map up to a constant scaling factor (24). In practice, variations in TOF resolution of detectors due to changes in the count rate can also impact the performance of TOF-MLAA. Moreover, accuracy of emission-based algorithms can be radiotracer dependent and sensitive to low counts. DL-based techniques, on the other hand, have either aimed to synthesize pseudo-CT images and denoise the MLAA μ-maps (25) or have attempted to perform simultaneous AC and SC in image space (26, 27) using deep convolutional neural network (CNN) architectures, such as U-Net (28) or Res-Net (29). Although promising results were obtained for standard-dose ^18^F-fluorodeoxyglucose (FDG) scans, these methods are also radiotracer dependent and could be prone to error with low-count data, radiotracers with very specific uptake, or in early frames of a dynamic scan when rapid changes of biodistribution can be expected with high-intensity local activity concentrations. Using the lutetium background radiation can theoretically address most of these shortcomings, if sufficient background counts are acquired, and, potentially, can be combined with both emission-based and DL-based methods for optimized performance.

Lutetium background radiation is present in all current-generation commercial PET scanners that use lutetium-based crystals [e.g., lutetium oxy-orthosilicate (LSO) and lutetium yttrium oxy-orthosilicate (LYSO)] for photon detection. The source of this background radiation is ^176^Lu, which is abundant in 2.6% of natural occurring lutetium and decays by β^−^ emission (99.66%), followed by one or more prompt gamma emissions at 307 keV (100%), 202 keV (83.3%), and 88 keV (15.5%) (30). The β^−^ often deposits its energy in the same crystal, and the gamma-rays can travel through the subject's body and get detected in an opposing crystal. Although the physics of lutetium background radiation is well understood (30, 31) and the idea of utilizing this radiation for PET AC and SC was first published in 2014 (32), there has been no direct implementation of this method in clinical or research settings. In previous studies with short-AFOV scanners (32–34), the MLTR reconstruction of the lutetium background could not provide adequate image quality to derive μ-maps without an increase in scan duration. Therefore, it was suggested that MLTR reconstructions of the lutetium background data can either be used only for 511 keV scatter estimation and initialization of the μ-maps in MLAA-like algorithms to jointly reconstruct the activity- and μ-maps (32), or the lutetium transmission data can be integrated in the objective function of the MLAA algorithm (34) while refraining from the known pitfalls (24, 35, 36) of the standard MLAA in finding a unique solution when PET data are used alone. While both approaches yield promising results, in absence of sufficient transmission counts, the performance of the algorithm strongly depends on the count statistics of the PET emission data and makes these methods vulnerable to radiotracer distribution and statistical errors in low-count PET data. Consequently, required scan duration per bed position and quantification uncertainties, due to insufficient lutetium background counts and contamination from standard-dose PET emission data on the μ-maps, were the main challenges that possibly prevented use of this method in clinical applications.

The latest generation of PET scanners with an increased AFOV has more crystal volume and, therefore, an increased flux of background radiation and substantially higher sensitivity for detecting it. This, in addition to possibility of acquiring images in a single bed position, enables effective use of the background radiation in applications that were not practically feasible with conventional scanners. Preliminary results on studying utilization of the lutetium background for AC in total-body PET were first presented in 2020 for the uEXPLORER (37) and the Biograph Vision Quadra (38) long-AFOV PET scanners. Furthermore, a simulation study on the uEXPLORER scanner was presented in 2021, showing promising preliminary results in using the lutetium-based μ-maps for PET motion correction (39). The most recent work presented on utilizing the lutetium background for AC was by Teimoorisichani et al., which included 3 patient scans on the Biograph Vision Quadra scanner (40). However, these results were obtained by performing two 5-min scans before and after the PET acquisition, and the reconstructions were performed with a limited acceptance angle of 18°, similar to a conventional 24-cm-long scanner. Therefore, MLTR reconstructions of the lutetium background still suffered from low count statistics of the lutetium background data and did not provide acceptable quantification accuracy for AC. Consequently, emission data from the ^18^F-FDG PET scans performed with ~3 MBq/kg injection of ^18^F-FDG (i.e., equivalent of ~211 MBq injection in a 70-kg patient) at 55–65-min post injection were used for AC in TOF-MLAA and TOF maximum likelihood estimation of activity and attenuation correction coefficients (MLACF) (41, 42) algorithms, initialized by the MLTR-based μ-maps, to improve the quantification accuracy.

In this work, utilization of lutetium background for AC will be studied in the uEXPLORER total-body PET scanner, with particular attention toward ultralow-dose PET scans that are now made possible with these long-AFOV scanners. Since acquiring the lutetium background data requires changes to the coincidence processor firmware on the clinical system, this work uses Monte-Carlo simulations of the scanner as a first step to understand the potential and requirements for future firmware modifications, algorithm developments, and data acquisition protocols. Additionally, Monte Carlo simulations provide the ground truth images for a quantitative analysis and can be used to provide one-to-one comparison between different scanner geometries. To our knowledge, this is the first study where lutetium-based AC is implemented in total-body PET reconstruction using large acceptance angles and the first time that it is used with ultralow-dose PET scans, where using MLAA-like algorithms becomes challenging due to the low-count nature of the PET emission data. Use of regularized MLTR algorithm for reconstructing the PET μ-maps only employing the lutetium transmission data is compared to reconstructions with MLAA using both TOF PET data and lutetium transmission data (MLAA-TX), which has been recently proposed by Cheng et al. (34) and tested with a short-AFOV scanner for a regular-dose PET scan. With the ultimate goal of understanding the requirements for using the lutetium background for AC in total-body PET, this paper will specifically investigate the effects from an increased acceptance angle, reduced scan duration, and Compton scattering on PET quantification; and will provide a comparison between two long-AFOV scanners (the uEXPLORER and a one-meter-long scanner) and a 24-cm-long conventional scanner.

## Materials and methods

### The uEXPLORER total-body PET/CT scanner

The uEXPLORER total-body PET/CT scanner was developed in a collaboration between University of California, Davis (UC Davis) and United Imaging Healthcare (UIH). The scanner has been fully described and characterized in two recent works (11, 43) and a summary of the system specifications is presented here. The uEXPLORER is composed of eight 24-cm-long PET units covering an axial length of 194 cm and a 160-slice CT scanner. Each PET detector block in the scanner consists of a 7 × 6 array of 2.76 × 2.76 × 18.1 mm^3^ Ce:LYSO scintillation crystals (Crystal Photonics Inc., Sanford, FL, United States), coupled to a 2 × 2 array of 6 × 6 mm^2^ silicon photomultipliers (SiPMs) (J-series, onsemi, Phoenix, Arizona, USA). The specifications of the scanner are listed in Supplementary Table 1. The coincidence detection is performed using an energy window of 430–645 keV and a variable coincidence window of 4.5–6.9 ns, which is defined based on a maximum-unit-difference (MUD) metric. The coincidence processing is performed with a MUD of 4, limiting the acceptance angle to 57° and uses a transverse reconstruction FOV of 600 mm.

### Monte Carlo simulations in GATE

The Geant4 Application for Tomographic Emission (GATE) v8.2 (44) with Geant4 v10.5.1 and ROOT v6.18/04 toolkits was used for Monte Carlo simulations of the uEXPLORER scanner. The detectors were simulated with an energy resolution of 11.7% at 511 keV energy and a coincidence time resolution of 430 ps. Singles were saved to file in the ROOT format. ^176^Lu activity in the scintillators was included in all simulations and was estimated from a 1-h blank scan on the uEXPLORER, yielding an average count rate of 92.2 cps per cc of LYSO in the scanner's default energy window (430–645 keV).

A 3D extended cardiac-torso (XCAT) voxelized phantom (45) defined based on GATE materials database was used for the simulations. The phantom represented a 58 y/o female (165-cm height, 69-kg weight), and the activity distribution of a healthy female subject scan on the uEXPLORER was scaled and used to simulate a 20-min ultralow-dose scan (37 MBq ^18^F-FDG injection, scanned at 90-min post injection). The total activity in the simulated phantom at scan time was 21 MBq. Twenty-two spherical lesions of different sizes (8–12 mm diameter) and contrasts (2.4–24 lesion-to-background ratios) were embedded in the phantom to study the effects on lesion quantification, which could be of particular interest in longitudinal studies, where CT scans may not be required at all time points, and quantification of small foci of uptake may be affected. This included 5 lesions in the liver, 3 lesions in each lung, 4 pelvic lymph nodes, 4 neck lymph nodes, and 3 lesions in the brain. The specifications of the lesions are provided in Supplementary Table 2, and the location of each lesion is shown in the transverse through the center of the lesion in Supplementary Figure 1.

A 200-min blank scan with the ^176^Lu activity was simulated for transmission scan reconstructions. Additionally, a 30-min scan of an annulus shell of ^18^F solution (with an outer diameter of 778 mm and an inner diameter of 772 mm, filled with activity concentration of 5.3 kBq.ml^−1^) was simulated to calculate PET normalization factors. Both datasets were smoothed in sinogram space by applying geometrical symmetries. Ground truth μ-maps at 511 keV were obtained by GATE's “MuMapActor” in all cases. The simulations were performed using an ion source definition both for ^18^F and ^176^Lu, in 50-ms time frames. Radioactive decay of ^18^F was accounted for during the generation of sinograms. The “emlivermore_polar” physics list from GATE was used for all the simulations.

To validate the GATE model for the scanner, a 20-min scan of the NEMA NU 2-2018 image quality phantom filled with ^18^F and a 10-min scan of a 5-MBq 170-cm-long line source (1-mm diameter) with back-to-back gamma emissions were additionally simulated and compared to the NEMA NU 2 experimental measurements on the physical scanner.

### Coincidence processing

ROOT singles were first converted into a 64-bit in-house singles format containing the eventID, the two crystals' transaxial and axial IDs, an energy tag representing predefined energy windows, sourceID, Compton scattering flag, and the time stamps saved with 39.0625 ps bin width, similar to the physical system. An in-house developed software-based coincidence processor (46) was then used for both emission data and lutetium transmission data. A 6.9-ns coincidence time window and the “takeAllGoods” coincidence policy were used to include the transmission coincidences. Randoms were discarded in all cases by identifying the “eventID” tag of the two singles. The 307-keV transmission coincidences were selected by using a 250–645 keV window for detecting the β^−^ energy deposition and a 250–350 keV window for detecting the 307 keV photons. The 511-keV emission coincidences were selected by using the 430–645 keV default energy window. Transmission and emission list-mode coincidences were converted into separate 4D and 5D sinograms, respectively, using an in-house developed C++ software tool. In all cases, unless otherwise stated, every three crystals in the axial direction were binned (mashed) together to reduce the sinogram size and, consequently, speed up the data processing and image reconstruction. To study the effects of Compton-scattered events on quantification of the μ-maps and activity maps correspondingly and to quantify the contamination of Compton-scattered emission data on transmission data in such ultra-low dose studies, Compton-scattered transmission events were differentiated from Compton-scattered emission events using the source position of the event and were included in the sinograms separately and together. To minimize the Compton-scattering contamination from the 511 keV data on 307 keV coincidences, only transmission coincidences with TOF larger than LOR's TOF threshold were accepted. The TOF threshold for each LOR was defined by subtracting 500 ps from the TOF corresponding to crystals' center-to-center distance of the LOR. In the reconstructions marked by “no scatter” in the Compton scattering section of the study and in all other sections of the study, Compton-scattered events were removed from the list-mode data, using the “comptonPhantom” tag of the ROOT Singles to exclusively compare the sensitivity gain on true coincidences, assuming that a scatter-correction method is implemented.

Sinograms with different acceptance angles and scan durations were generated from the uEXPLORER's simulated coincidence data to represent 20-min whole-body scans on three scanner geometries: the 194-cm-long uEXPLORER, a one-meter-long scanner (using 4 uEXPLORER axial units), and a 24-cm-long conventional scanner (using one uEXPLORER axial unit). The 20-min total-body scan on the uEXPLORER used a single bed position, the one-meter-long scanner was simulated with two 50%-overlapping bed positions (10 min per position), and the 24-cm-long conventional scanner was simulated with eight bed positions (2.5 min per position) in order to scan head-to-thighs on all three scanners. While the uEXPLORER coincidence processing was performed with a MUD of 4, no axial unit-difference policy was used for the two shorter scanner geometries, and coincidences between all crystals rings within the scanner length were accepted. Maximum acceptance angles were 57°, 51°, and 17° for the uEXPLORER, one-meter-long, and 24-cm-long scanners, respectively. The scan start positions for the two shorter scanners were adjusted to achieve high sensitivity in the brain.

To study the effect of transmission scan duration on accuracy of the μ-maps, sinograms for scan durations of 5 min, 10 min, and 20 min were generated and reconstructed. Furthermore, to investigate the transmission sensitivity gain by an increased acceptance angle, the uEXPLORER data with a maximum acceptance angle of 57° and MUD 4 were additionally compared to maximum acceptance angles of 51°, 43°, 32°, and 17°, corresponding to 4 to 1 uEXPLORER axial unit lengths. Finally, to assess the effect of Compton scattering on accuracy of the μ-maps, the Compton scattered 307 keV photons and 511 keV photons were added to the sinograms separately and together, with and without the LOR TOF validation, and, additionally, with using an energy threshold of 290 keV instead of 250 keV for the two energy windows of the transmission scans (i.e., 290–350 keV for the 307 keV photons and 290–645 keV for the β^−^).

### Image reconstruction

An in-house developed image reconstruction framework, including TOF-MLEM, MLTR, and MLAA-TX algorithms with an extended separable quadratic surrogate (SQS) update (34), was used. All reconstruction algorithm implementations were sinogram based and were accelerated by ordered subsets. The framework was specifically designed to allow parametrized cylindrical geometry definition and to support total-body PET reconstruction. The codes were written in C/C++ and used CUDA to allow the majority of computationally expensive functions to run on graphics processing units (GPUs). A Linux computational node equipped with two 12-core Intel (R) Xeon (R) Gold 6126 central processing units (CPUs), 1.48 TB memory, an NVIDIA Tesla V100 PCIe GPU (with 32 GB memory), and 48 TB hard disk storage was used for a single reconstruction task.

The μ-maps were first reconstructed with a penalized MLTR algorithm using a separable quadratic surrogate update function (34) and a quadratic regularization. The MLTR-based μ-maps were then used first directly for AC in the TOF-MLEM algorithm and, second, as an initialization for the MLAA-TX algorithm. The MLTR updates in the MLAA-TX algorithm also included a quadratic regularization. All reconstructions were performed with 4-mm isotropic voxels. For the uEXPLORER and the one-meter-long scanner, reconstructions were performed with 10 non-overlapping subsets, ordered by projections equidistant in angle; and for the 24-cm-long scanner 4 non-overlapping subsets were used due to lower sensitivity of the scanner.

Similar to the standard TOF-MLAA algorithm that uses an interleaved updating method at each iteration, the MLAA-TX algorithm first calculates the sensitivity map from the available μ-map, μ, and updates the activity map, λ, using the TOF-MLEM algorithm while keeping the μ-map constant, and subsequently updates the μ-map. In contrast to the standard TOF-MLAA in which TOF emission data are solely used for updates of the activity and μ-maps, the MLAA-TX algorithm combines the TOF emission data and transmission data with a weighting factor, α, to update the μ-map. Availability of TOF information results in a faster convergence for the TOF-MLEM algorithm. Therefore, every iteration of the MLAA-TX algorithm included one update of the activity map and five updates (iterations) of the μ-map. The update of the sensitivity map and the activity map with the TOF-MLEM algorithm can be written as:


$$\begin{array}{l} \forall j:\lambda_{j}^{\left(n+1\right)} \end{array}$$

(1) $$\begin{array}{l} =\frac{\lambda_{j}^{\left(n\right)}}{\sum_{i=1}^{M}p_{ij}a_{i}^{\left(n\right)}n_{i}}\underset{it}{\sum }p_{itj}\frac{a_{i}^{\left(n\right)}n_{i}y_{it}}{a_{i}^{\left(n\right)}n_{i}\sum_{j=1}^{N}p_{itj}\lambda_{j}^{\left(n\right)}+s_{it}}, \end{array}$$

(2) $$\begin{array}{l} \forall i:a_{i}^{\left(n\right)}=e^{-\sum_{j=1}^{N}p_{ij}\mu_{j}^{\left(n\right)}} \end{array}$$

where${\;\lambda }_{j}^{\left(n\right)}$ denotes the value of voxel *j* of the reconstructed activity image λ at iteration *n*, *y*_*it*_ is the measured counts for TOF bin *t* of LOR *i*, *s*_*it*_ is the expected contribution from scatter and/or randoms, and *p*_*itj*_ is the TOF system matrix element representing the probability of detection in TOF bin *t* of LOR *i* for an emission in voxel *j*. $a_{i}^{\left(n\right)}$ represents the attenuation factor for LOR *i* at iteration *n*, which is calculated from forward projection of the μ-map μ^(*n*)^, using non-TOF system matrix elements *p*_*ij*_. The value of voxel *j* of the sensitivity map is calculated by the denominator term $\overset{M}{\underset{i=1}{\sum }}p_{ij}a_{i}^{\left(n\right)}n_{i}$, where *n*_*i*_ denotes the normalization factor for LOR *i*. A component-based iterative normalization method (47) was used to estimate the normalization factor *n*_*i*_ for each LOR. TOF-MLEM reconstructions were performed with TOF bin width of 273 ps. No point spread function (PSF) modeling was included in the reconstructions.

The update of the activity map is subsequently followed by the update of the μ-map. The forward projection of the updated activity map denoted by $\phi_{i}^{\left(n+1\right)}$and the forward projection of the current μ-map denoted by $z_{i}^{\left(n\right)}$are defined to simplify the subsequent update equations as follows:


(3) $$\begin{array}{lll} \forall i:\phi_{i}^{\left(n+1\right)} & = & \underset{j}{\sum }p_{ij}\lambda_{j}^{\left(n+1\right)}, \end{array}$$

(4) $$\begin{array}{lll} \forall i:z_{i}^{\left(n\right)} & = & \underset{j}{\sum }p_{ij}\mu_{j}^{\left(n\right)}. \end{array}$$

The update of the μ-map with the extended SQS method is performed by maximizing an objective function *L*_*MLAA*−*TX*_(λ, μ) defined based on Poisson log-likelihood objective functions *L*_*emis*_(λ, μ) and *L*_*tran*_(μ_*tran*_) for emission data and transmission data, respectively, and the image roughness penalty *U*(μ) used with the regularization parameter β:


(5) $$\begin{array}{l} L_{MLAA-TX}\left(\lambda ,\mu \right)=L_{emis}\left(\lambda ,\mu \right)+\alpha L_{tran}\left(\mu_{tran}\right)-\beta U\left(\mu \right), \end{array}$$

$$\begin{array}{l} ≜-\sum_{i}h_{i}(z_{i}^{\left(n\right)})-\alpha \sum_{i}H_{i}(Z_{i}^{\left(n\right)})\beta \underset{j}{\sum }\underset{k\in N_{j}}{\sum }\\ \frac{1}{4}w_{jk}{\left(\mu_{k}^{\left(n\right)}-\mu_{j}^{\left(n\right)}\right)}^{2} \end{array}$$

where


(6) $$\forall i:h_{i}\left(z_{i}^{\left(n\right)}\right)\text{ ​}=\left(\text{​}\phi_{i}^{\left(n+1\right)}e^{-z_{i}^{\left(n\right)}}\text{​}+\text{​}s_{i}\right)\text{​}-\text{​}y_{i}ln\left(\text{​}\phi_{i}^{\left(n+1\right)}e^{-z_{i}^{\left(n\right)}}+s_{i}\text{​}\right)\text{​},$$

(7) $$\forall i:H_{i}\left(Z_{i}^{\left(n\right)}\right)=\left(\text{​}B_{i}e^{-Z_{i}^{\left(n\right)}}\text{​}+\text{​}r_{i}\right)\text{​}-\text{​}y_{tran,i}ln\left(\text{​}B_{i}e^{-Z_{i}^{\left(n\right)}}\text{​}+\text{​}r_{i}\text{​}\right),$$

and


(8) $$\begin{array}{l} \forall i:Z_{i}^{\left(n\right)}=\underset{j}{\sum }l_{ij}\mu_{tran,j}^{\left(n\right)}. \end{array}$$

The quadratic function of the intensity difference between the neighboring voxels has been used for the penalty function, in which the *N*_*j*_ represents a collection of 26 closest voxels in the neighborhood of voxel *j*, and *w*_*jk*_ is an inverse-distance weighting factor assigned to each neighbor voxel *k*. The μ_*tran*_ represents the linear attenuation coefficients at 307 keV and is estimated by a linear transformation μ_*tran*_ = ημ with η= 1.2276. The η value was estimated by linear fitting of the attenuation coefficients of different tissues in the human body (ranging from air to the bone) at 307 and 511 keV, using the GATE materials database. From the transmission data, *B*_*i*_ denotes the number of transmission counts recorded for LOR *i* in the blank scan, *y*_*tran, i*_ represents the number of transmission counts measured for LOR *i* in presence of the subject, *r*_*i*_ is the expected contribution from scatter and/or randoms in the transmission data, and *l*_*ij*_ is the system matrix element for transmission data. The update of the μ-map in MLAA-TX with the extended SQS method (34) and the quadratic penalty (32) can be written as:


(9) $$\forall j:\mu_{j}^{\left(n+1\right)}=\mu_{j}^{\left(n\right)}-\frac{\sum_{i}l_{ij}(\dot{h_{i}}(z_{i}^{\left(n\right)})+\alpha \eta \dot{H_{i}}(Z_{i}^{\left(n\right)}))-\beta \sum_{k\in N_{j}}w_{jk}(\mu_{k}^{\left(n\right)}-\mu_{j}^{\left(n\right)})}{\sum_{i}l_{ij}({\overset{ˇ}{c}}_{i,emis}^{\left(n\right)}+\alpha \eta^{2}{\overset{ˇ}{c}}_{i,tran}^{\left(n\right)})L_{i}+\beta \sum_{k\in N_{j}}w_{jk}},$$

where


(10) $$\begin{array}{l} \forall i:L_{i}=\underset{k}{\sum }l_{ik} \end{array}$$

and ${č}_{i,emis}^{\left(n\right)}$ and ${č}_{i,tran}^{\left(n\right)}$ are curvature terms for emission data and transmission data, respectively, defined in (34).

The update of the μ-map in the MLTR algorithm was performed in a similar way by only employing the transmission data. The system matrix was calculated on the fly in all cases based on the Siddon ray tracing algorithm (48, 49). Although the reconstruction algorithm allowed random sampling of the two LOR end-point positions within the crystals with an exponential probability function defined along crystal length, the endpoint positions were assigned to the central coordinates of the crystal surface plane and at ~7-mm crystal depth in all cases to speed up the reconstructions in this work, at the expense of increased noise. The probability function in the projector was separately tuned for 307 keV and 511 keV photons, in which the interaction distance from the front surface of the crystal was modeled as (50):


(11) $$\begin{array}{l} \frac{\ln\left(1-Y\left(1-exp\left(-{\eta \mu }_{LYSO}D\right)\right)\right)}{-\eta \mu_{LYSO}} \end{array}$$

where *Y* was a random number between 0 and 1, set to 0.6 when random sampling was disabled, μ_*LYSO*_ was the linear attenuation coefficient of the LYSO crystal at 511 keV energy, set to 0.0815 mm^−1^ derived based on GATE simulations, and D was the crystal length set to 18.1 mm. η = 1.2276 was used for the transmission system matrix for the 307 keV energy and η was set to 1 for PET 511 keV system matrix. *Y* = 0.6 was chosen by analyzing the mean interaction depths in the GATE simulations. The regularization parameter β was tuned for each scanner geometry by scanning a range of values varying from 4,000 to 100,000.

To mitigate the effect of Compton scattering on quantification accuracy of the μ-maps and activity maps consequently, the MLTR-reconstructed μ-maps that included Compton-scattered events were scaled with a global scaling factor similar to the approach proposed by (40). This was done by finding the water peak in the histogram of the image voxel values of the μ-maps and computing a global scaling factor to shift the histogram peak to the attenuation coefficient of water.

### Image quality analysis

Image quality analysis was performed on reconstructed images from all iterations in MATLAB. Quantification accuracy assessment was performed by voxel-wise comparison of reconstructed images to ground truth simulated images, both for activity maps and μ-maps, using normalized root mean square error (NRMSE) and structural similarity index (SSIM) as figures of merit. NRMSE for image *x* was defined in reference to ground truth image $\;\overset{ˇ}{x}$ as:


(12) $$NRMSE=\frac{RMSE}{\underset{j}{\max}x_{j}-\underset{j}{\min}x_{j}}=\frac{\sqrt{\frac{\sum_{j=1}^{N}{\left(x_{j}-{\overset{ˇ}{x}}_{j}\right)}^{2}}{N}}}{\underset{j}{\max}x_{j}-\underset{j}{\min}x_{j}}$$

and SSIM was used with equal weighting for luminance, contrast, and structural terms. The optimal iteration number, regularization parameter, and the MLAA-TX weighting factor were chosen for each scanner geometry's reconstruction by maximizing the SSIM and minimizing the NRMSE. In a few cases where SSIM, and NRMSE-optimized parameters did not agree, the regularization parameter minimizing the NRMSE was selected. Unless otherwise stated, MLTR reconstructions at iteration 50 were chosen for all three scanners; for MLEM and MLAA-TX reconstructions, iteration 3 was chosen for the uEXPLORER geometry and iteration 2 was used for the one-meter and 24-cm geometries; and β regularization parameters of 10,000, 20,000, and 80,000 were used for the uEXPLORER, one-meter, and 24-cm geometries, respectively. MLAA-TX reconstructions with weighting factors (α) of 0.1, 1, and 10 were reconstructed for all three scanner geometries and based on the SSIM and NRMSE comparisons α = 1 was used for comparison of the three scanners. At the chosen iteration and regularization parameter, bias-variance trade-off was assessed by placing 23 spherical volumes-of-interest (VOIs) on selected anatomical regions. Size, location, and mean ground truth activity concentrations of the VOIs are specified in Supplementary Table 3 and shown in Supplementary Figure 2. The voxel-wise bias was calculated for each voxel *j* of image *x* in reference to the voxel values in the ground truth-simulated image$\;\overset{ˇ}{x}$ as:


(13) $$Bias_{j}=\frac{x_{j}-{\overset{ˇ}{x}}_{j}}{{\overset{ˇ}{x}}_{j}}$$

and bias distribution was compared for the voxels contained in the VOIs for each organ using box and whiskers plots. Finally, lesion quantification in the reconstructed activity maps was assessed by visual comparison of transverse slices, drawing line profiles through the center of each lesion, and calculating the SUV_max_ percentage bias for each lesion, calculated in reference to the ground truth activity map and the OSEM-reconstructed activity map using the ground truth μ-map. The reference OSEM reconstructions using the ground truth μ-map were done separately for each scanner geometry.

## Results

### Monte-Carlo model validation

**Figure 1:**
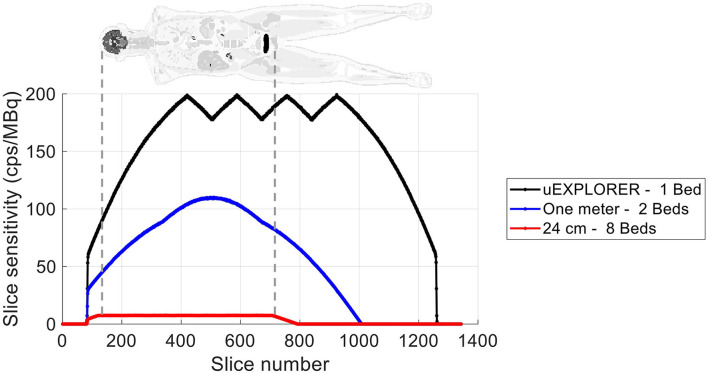
The axial sensitivity profile at the center of the FOV, calculated from the 170-cm-long line source simulation, compared for the three scanner geometries. The position of the XCAT phantom is shown additionally as a reference.

The axial sensitivity profiles at the center of the FOV are shown for the three scanners in Figure 1, using the 170-cm-long line source simulation. The position of the simulated XCAT phantom is also shown relative to the sensitivity profile as a reference. The total sensitivity of the simulated uEXPLORER geometry with the 170-cm line source was 190 kcps/MBq, and peak SSRB slice sensitivity was 199 cps/MBq. For comparison, the measured total sensitivity of the uEXPLORER scanner with the 170-cm line source was 147 kcps/MBq, and peak SSRB slice sensitivity was 158 cps/MBq (11).

The reconstructed image and the results of the NEMA NU 2 image quality analysis of contrast recovery and background variability on the simulated image quality phantom are shown in Supplementary Figure 3 for reconstructed images at iterations 3 and 10. Comparing the contrast recovery coefficients at iteration 3 to iteration 10 shows negligible improvements, suggesting that the algorithm has reached convergence. Using 2.344-mm isotropic voxels and 3 iterations (10 subsets), the contrast recovery coefficient varied from 62% for the smallest sphere to 91% for the largest sphere; and the background variability was 2.6% to 0.6%, respectively. No axial mashing was used for the reconstructions in this step to not introduce additional blurring and contrast loss. For comparison, the measured contrast recovery coefficients of a 30-min scan of the NEMA NU 2 image quality phantom reconstructed with the vendor's reconstruction software with 2.344-mm isotropic voxels and 4 iterations (20 subsets) and no PSF modeling were 50% and 91% for the smallest and largest spheres, respectively; and the background variability was 3.1% to 1.3%, respectively (11).

The energy spectra of the singles detected from the ^18^F emissions of the simulated XCAT phantom and the lutetium background emissions in the uEXPLORER geometry are shown in Supplementary Figure 4 individually and all together. The emission peaks at 202 keV, 307 keV, and 511 keV are visible in addition to the contributions from Compton-scattered events.

### Effect of an acceptance angle on lutetium-based μ-maps

**Figure 2:**
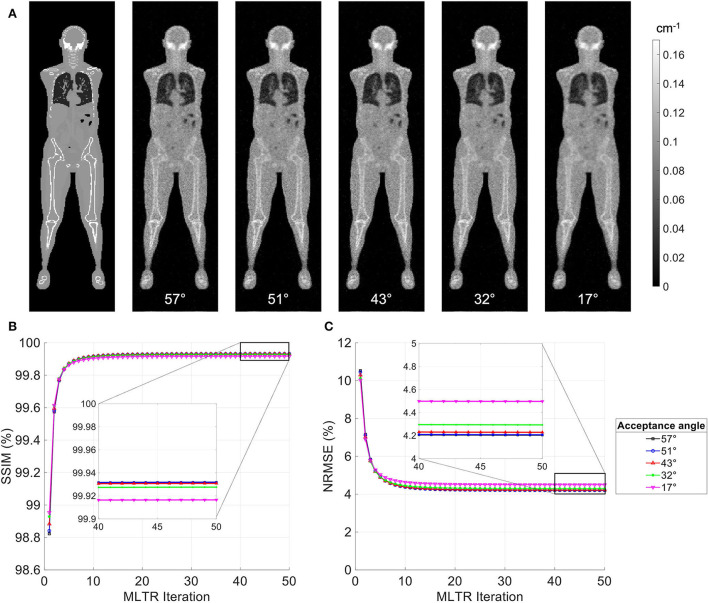
**(A)** Selected coronal slices from MLTR reconstructions of a 20-min lutetium transmission scan on the uEXPLORER, using maximum acceptance angles ranging from 57° to 17°, compared to the ground truth simulated μ-map. **(B)** SSIM and **(C)** NRMSE of the reconstructed images shown in percentages as a function of MLTR iteration.

MLTR reconstructions of a 20-min lutetium transmission scan on the uEXPLORER, using maximum acceptance angles ranging from 57° to 17° are shown in Figure 2, with corresponding SSIM and NRMSE values plotted for all 50 iterations. All reconstructions used a regularization parameter β = 10, 000. The boxplots of the bias distribution in selected organs of interest, calculated from VOI analysis on the μ-maps, are additionally shown in Supplementary Figure 5. As can be observed in the SSIM, NRMSE, and bias plots, there is negligible difference in image quality and quantification accuracy of the μ-maps reconstructed with acceptance angles higher than 32°. The mean bias in all organs of interest is close and within ± 10%. However, reducing the acceptance angle further to 17° affects the quantification accuracy of the attenuation coefficients, particularly in the bone and the bone marrow. To exclude the effect of regularization on bias measurements, reconstructed μ-maps with no regularization and their corresponding bias distributions are additionally shown in Supplementary Figures 6, 7, respectively. As expected, in absence of regularization, larger variations are observed in all organs due to increased noise. However, mean biases are still within ± 10% in all cases, except for biases in the bone marrow and lungs when the acceptance angle is reduced to 17°.

### Effect of scan duration on lutetium-based μ-maps

**Figure 3:**
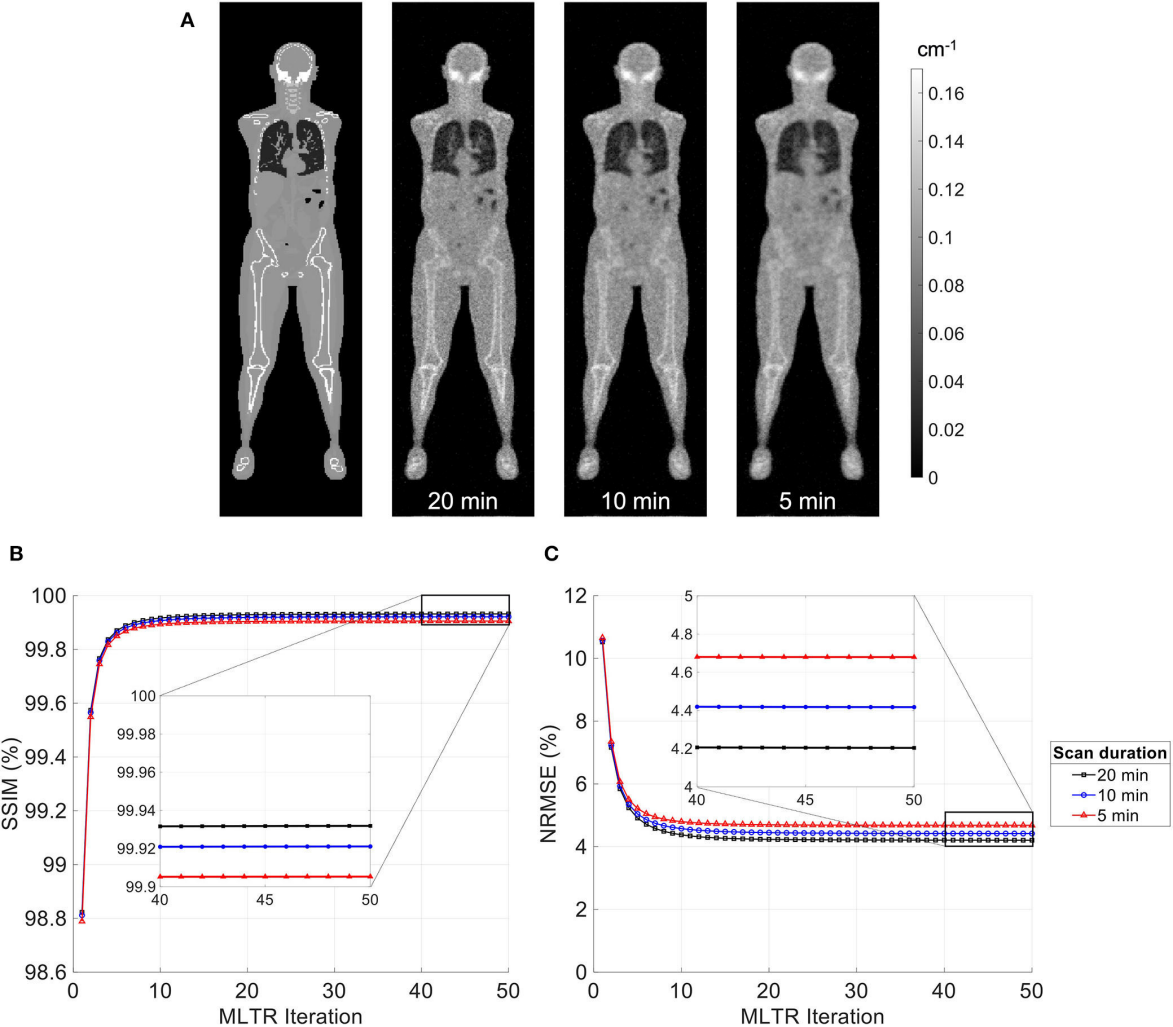
**(A)** Selected coronal slices from MLTR reconstructions of a lutetium transmission scan on the uEXPLORER, with scan durations of 5 min, 10 min, and 20 min, compared to the ground truth simulated μ-map. **(B)** SSIM and **(C)** NRMSE of the reconstructed images shown in percentages as a function of MLTR iteration.

MLTR reconstructions of lutetium transmission scans on the uEXPLORER, with scan durations of 5 min, 10 min, and 20 min, are shown in Figure 3, with corresponding SSIM and NRMSE values of all 50 iterations. A regularization parameter β of 40,000, 20,000, and 10,000 was used for the 5-min, 10-min, and 20-min scans, respectively. The box plots of the bias distribution in the selected organs of interest are additionally shown in Supplementary Figure 8. Reducing the transmission scan duration shows larger effects on the image quality and quantification accuracy of the μ-maps, compared to reducing the acceptance angle. However, while using a 10-min scan still yields mean biases within ±10% in all organs of interest, further reduction of the scan duration to 5 min results in increased biases beyond ±10% in bone structures and lungs. To exclude the effect of regularization on bias measurements, reconstructed μ-maps with no regularization and their corresponding bias plots are additionally shown in Supplementary Figures 9, 10, respectively. With no regularization, mean bias is still within ±10% in all cases, expect for the bias in the lungs when a 5-min-long scan is used.

### Lutetium-based AC in different scanner geometries

**Figure 4:**
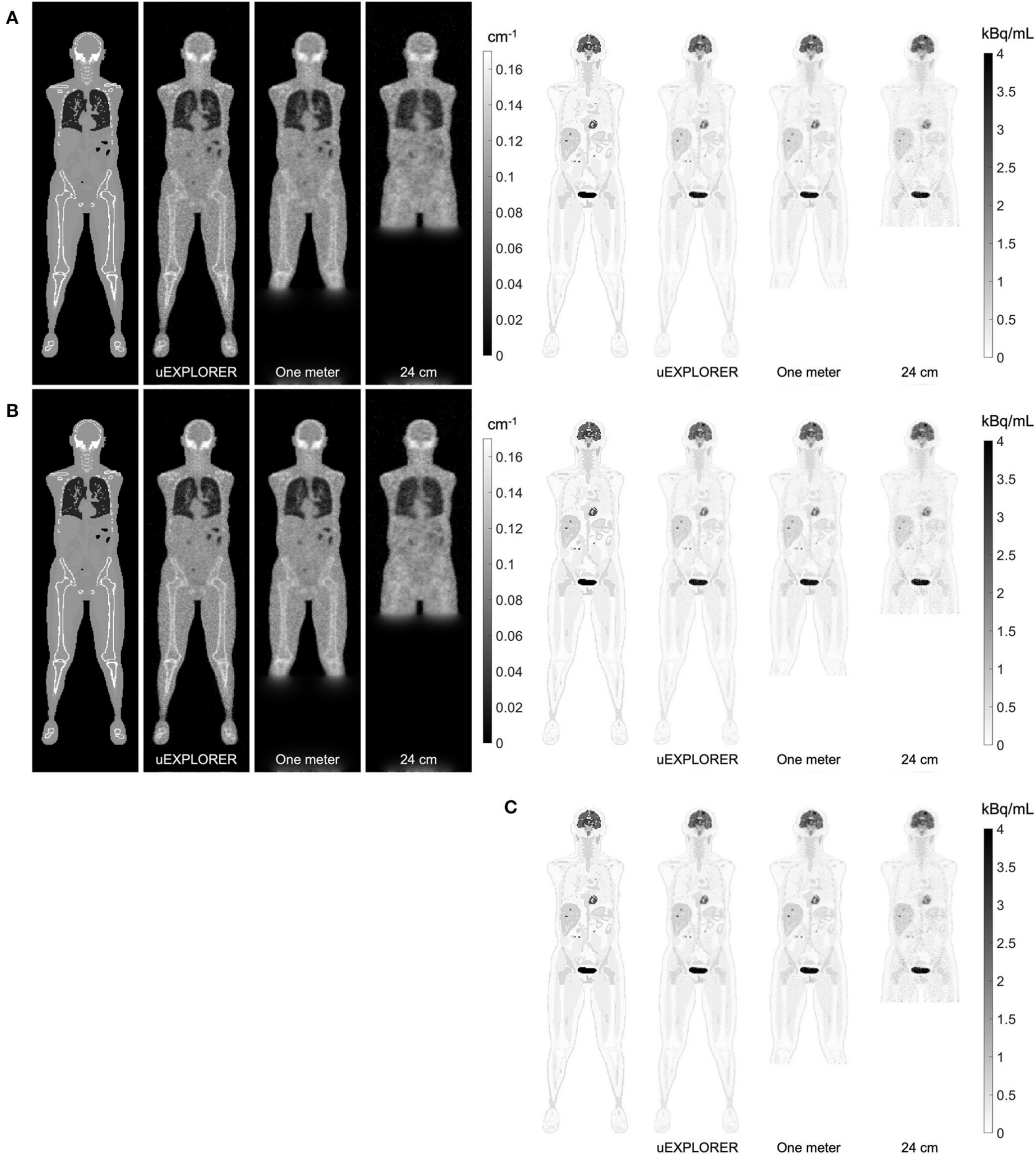
Selected coronal slices for a 20-min whole-body scan (37 MBq ^18^F-FDG injection, scanned at 90-min p.i.) on the three scanner geometries obtained from **(A)** MLTR reconstructions, and OSEM reconstructions of the activity maps using regularized MLTR-based μ-maps, **(B)** regularized MLAA-TX reconstructions of activity and μ-maps initialized with regularized MLTR reconstructions, and **(C)** OSEM reconstructions using ground truth μ-maps.

**Figure 5:**
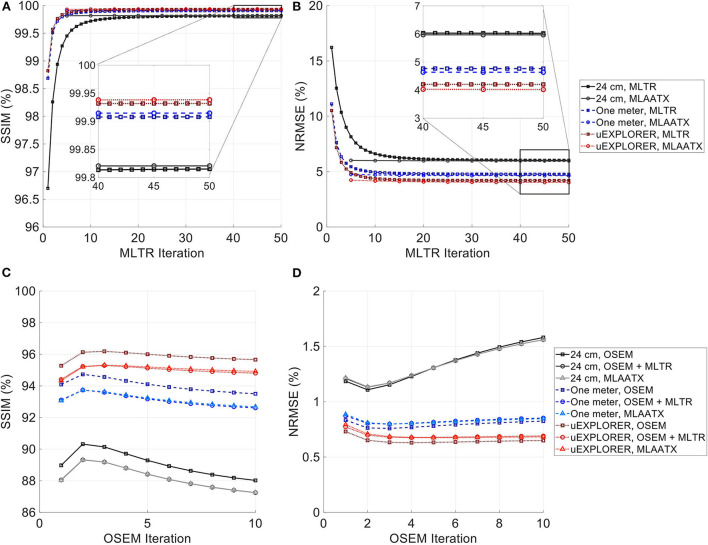
**(A,C)** SSIM and **(B,D)** NRMSE of the reconstructed **(A,B)** μ-maps and **(C,D)** activity maps compared for MLTR and MLAA-TX reconstructions with the three scanner geometries. The activity images are additionally compared to OSEM reconstructions using the ground truth μ-maps, plotted with square markers.

**Figure 6:**
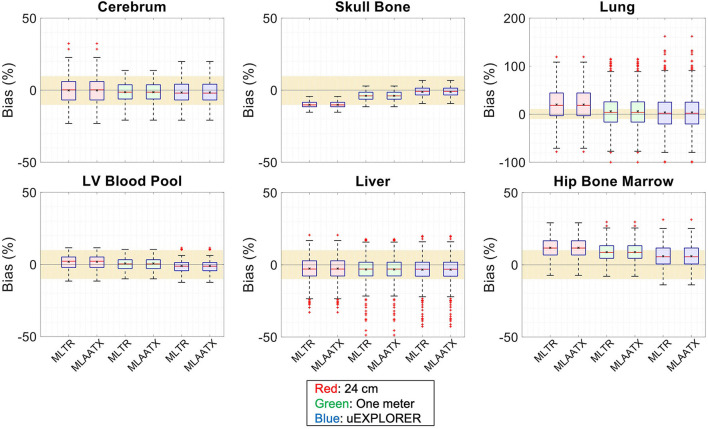
Attenuation map bias (%) distribution in selected organs of interest compared for the (red) 24-cm, (green) one-meter, and (blue) uEXPLORER scanners, using MLTR and MLAA-TX reconstructions with regularization. The region marked with a yellow color depicts the ±10% range. Note: All box plots share the same labels. Results for the lungs are shown on a different y-axis range due to higher variability.

**Figure 7:**
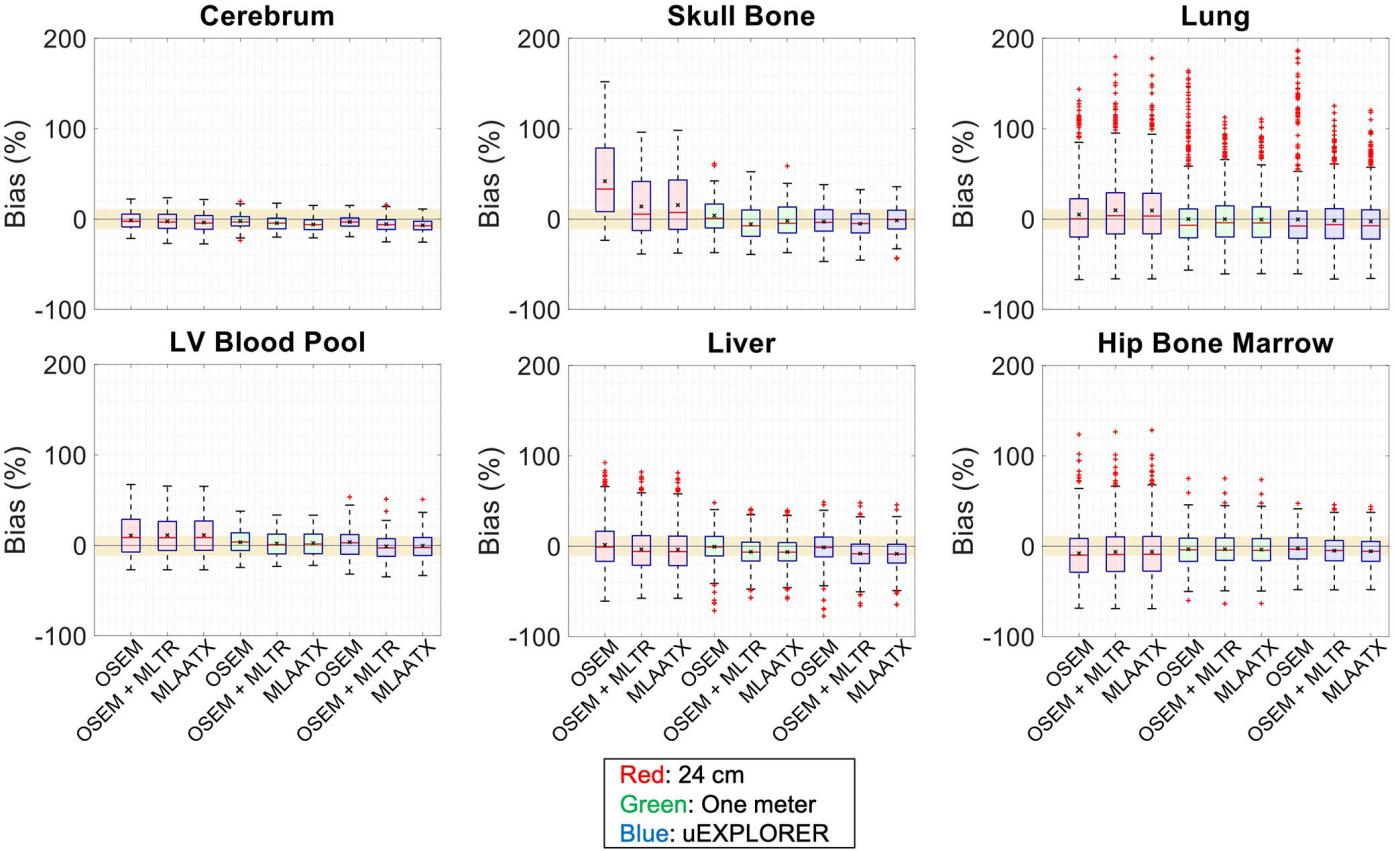
Activity map bias (%) distribution in selected organs of interest compared for the (red) 24-cm, (green) one-meter, and (blue) uEXPLORER scanners, using the OSEM with the ground truth μ-map, OSEM with the MLTR-based μ-map with regularization, and MLAATX with regularization. The region marked with a yellow color depicts the ±10% range. Note: All box plots share the same labels.

Figure 4 compares selected coronal slices of reconstructed attenuation and activity maps for a 20-min whole-body scan on three scanner geometries obtained from (1) regularized MLTR reconstructions of the μ-maps and OSEM reconstructions of the activity maps using MLTR-based μ-maps and (2) regularized MLAA-TX reconstructions of activity and μ-maps initialized with regularized MLTR reconstructions. OSEM reconstructions using ground truth μ-maps are additionally shown as a reference. Furthermore, the results of SSIM and NRMSE analysis on the images are shown in Figure 5, and the bias distribution in selected organs of interest is shown for the μ-maps and the activity maps in Figures 6, 7, respectively.

As expected, the uEXPLORER geometry offers improvements in SSIM and NRMSE of both μ-maps and activity maps, compared to the other two shorter geometries. However, the performance differences are much smaller between the uEXPLORER and the one-meter-long scanner than the differences between the 24-cm-long scanner and the two longer scanner geometries. The bone structures of the rib cage, the pelvis region, and the legs are particularly better recovered in the μ-maps of the uEXPLORER. Since the MLTR-based μ-maps are used for initialization of the MLAA-TX reconstructions, the convergence is accelerated in the MLAA-TX reconstructions, which can be observed in comparison of the earlier iterations. However, iterating longer with MLAA-TX seems to offer no substantial improvements in SSIM and NRMSE of the μ-maps compared to MLTR reconstructions. This is consequently reflected in comparison of the activity maps, where MLAA-TX images show only negligible improvements in SSIM and NRMSE. The difference between the two long-AFOV scanners and the 24-cm scanner becomes larger when comparing the activity maps of the ultralow-dose XCAT phantom. Using the scatter-free lutetium-based μ-maps for AC led to ~1% relative reduction of SSIM of the activity maps in all three scanner geometries (from 95.66%, 93.5%, and 88.02% using the ground truth activity maps to 94.81%, 92.61%, and 87.25% for the uEXPLORER, one-meter, and 24-cm scanners, respectively). Comparing the μ-maps reconstructed by MLAA-TX and MLTR also shows some transmission-emission crosstalk artifacts in high-activity regions, particularly visible in the bladder.

Due to the use of regularization, the noise properties of the μ-maps do not reflect the sensitivity differences and the μ-maps of the 24-cm-long scanner have been affected more by the regularization. Therefore, as a reference, MLTR and MLAA-TX reconstructions of the μ-maps with no regularization are shown with their corresponding activity maps in Supplementary Figure 11. Although MLAA-TX reconstructions were initialized with the regularized MLTR reconstructions in this case, they still suffer from increased noise in absence of regularization during the MLTR updates. The increased noise in the μ-maps also propagates into the activity maps of the three scanner geometries, particularly affecting the 24-cm-long scanner. To exclude the effect of regularization on bias calculations, the bias distributions of the reconstructions of the μ-maps with no regularization and their corresponding activity maps are shown in Supplementary Figures 12, 13, respectively.

Comparing the bias distribution of the μ-maps in Figure 6 shows that the mean bias in all organs of interest is within ±10% for the uEXPLORER and the one-meter-long scanner, while higher biases can be observed in attenuation coefficients of lungs and bone structures in the 24-cm scanner. Comparing these results to the bias distributions with no regularization in Supplementary Figure 12 shows that the high bias in the attenuation coefficient of the skull bone in the 24-cm-long scanner is partially caused by the regularization. However, not using the regularization introduces larger biases in the lungs and the hip bone marrow in the 24-cm-long scanner. Comparing the MLTR and MLAA-TX reconstructions shows negligible difference between the two methods in all three scanner geometries.

Bias distribution of the activity maps in Figure 7 also shows similar performance between the uEXPLORER and the one-meter-long scanner with biases within ±10% in all organs of interest. The 24-cm-long scanner's quantification is affected in bones and lungs. Comparing these results to the activity map biases in Supplementary Figure 13, which used μ-maps with no regularization, shows increased biases in lungs, bones, and left ventricle bloodpool. In all cases, negligible difference is observed between AC performed with MLTR and MLAA-TX μ-maps, except for slight improvement in the skull bones offered by MLAA-TX.

**Figure 8:**
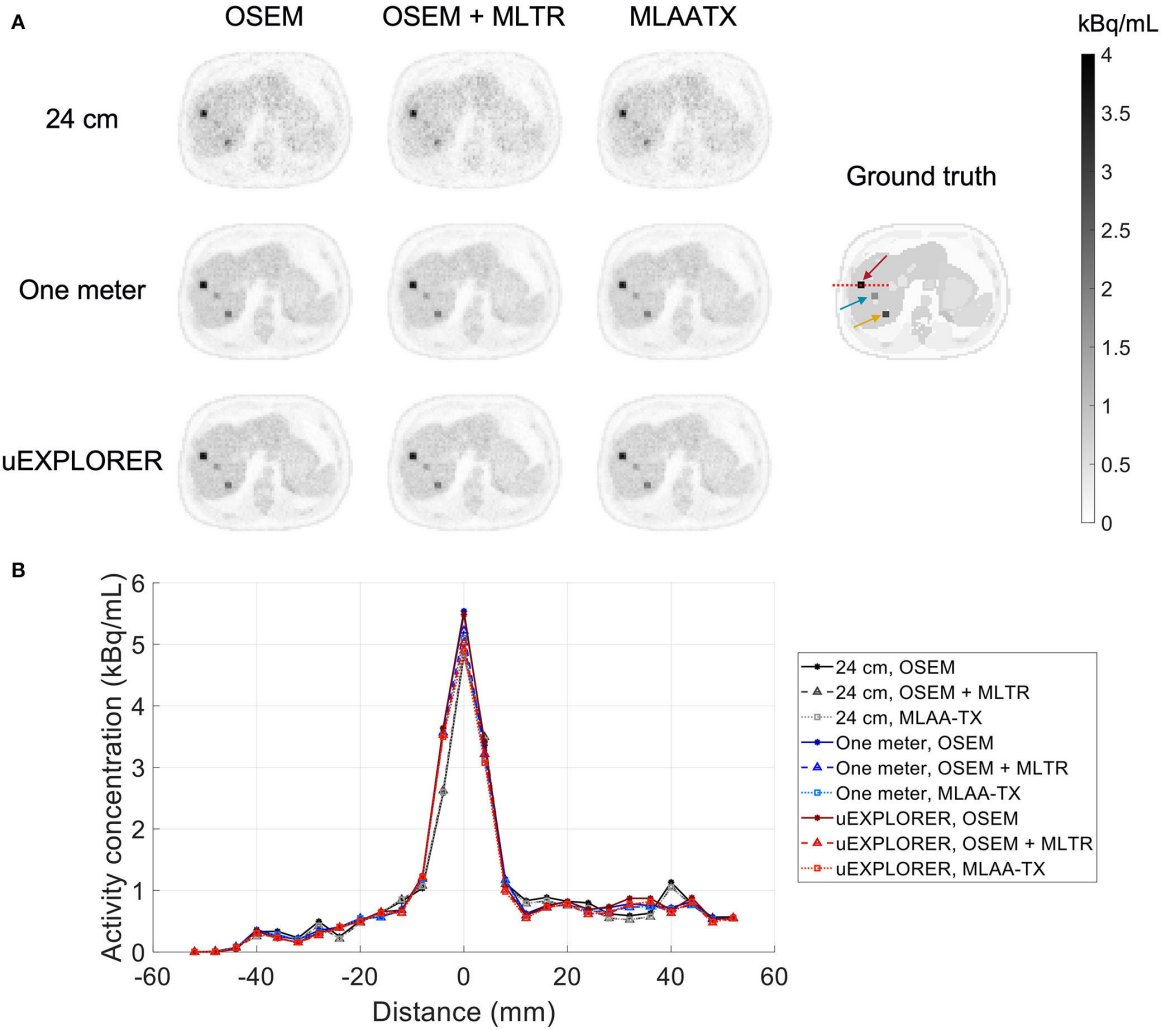
Comparison of lesion contrast in the liver, **(A)** showing 3 lesions in a transverse slice through the liver, reconstructed using OSEM with the ground truth μ-maps, OSEM with the regularized MLTR μ-maps, and regularized MLAA-TX, compared for the three scanner geometries and the ground truth activity map; and **(B)** 10-cm-long line profiles drawn horizontally through the center of the hottest lesion. The hottest lesion and the line profile location are marked with a red arrow and a red dashed line on the ground truth image, respectively. The two other lesions are marked with blue and yellow arrows.

**Figure 9:**
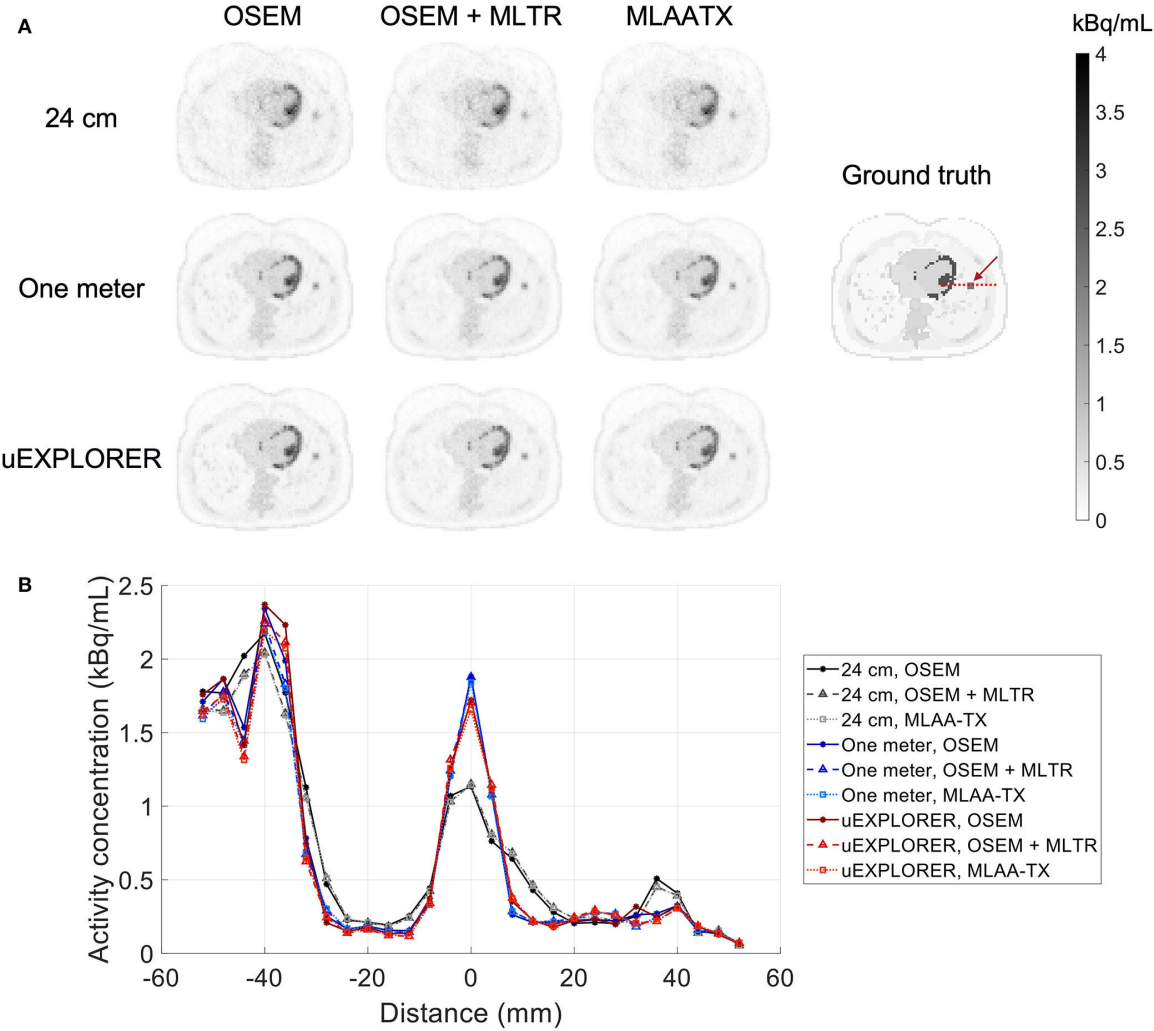
Comparison of lesion contrast in the lung, **(A)** showing an 8-mm lesion in a transverse slice through the lung reconstructed using OSEM with the ground truth μ-maps, OSEM with the regularized MLTR μ-maps, and regularized MLAA-TX, compared for the three scanner geometries and the ground truth activity map; and **(B)** 10-cm-long line profiles drawn horizontally through the center of the lesion. The lesion and the line profile location are marked with a red arrow and a red dashed line on the ground truth image, respectively.

**Figure 10:**
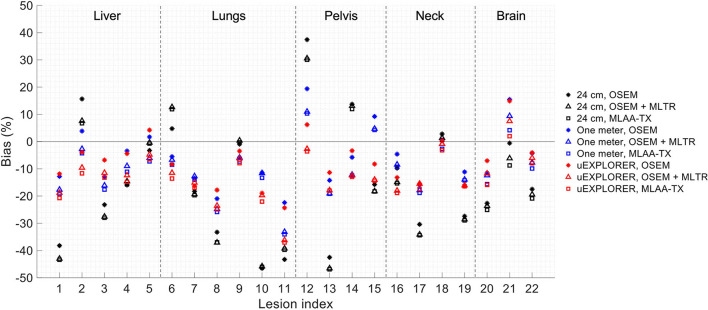
Percentage bias of SUV_max_ in 22 lesions of the XCAT phantom calculated in reference to the ground truth activity maps, compared for the OSEM-reconstructed images using the regularized MLTR μ-maps and regularized MLAA-TX reconstructions, shown for the three scanner geometries.

To show the effect of using the lutetium-based μ-maps on lesion quantification, two examples are shown in Figures 8, 9 for liver and lung lesions, respectively, with line profiles passing through the lesion center. The transverse slice through liver includes three lesions with different contrasts (lesions 2, 4, and 5 in Supplementary Table 2) and an 8-mm lesion (lesion 10 in Supplementary Table 2) was chosen as an example in the lung. Line profiles through all 22 lesions are additionally shown in Supplementary Figure 14. Furthermore, Figure 10 shows the percentage bias of SUV_max_ in all 22 lesions of the XCAT phantom calculated in reference to the ground truth activity maps, and Supplementary Figure 15 shows the percentage bias of SUV_max_ in the 22 lesions calculated in reference to the images reconstructed using the OSEM algorithm with the ground truth μ-maps. Comparing the activity maps reconstructed using the ground truth μ-maps to activity maps using the μ-maps reconstructed with regularized MLTR and MLAA-TX algorithms in Figures 8, 9, there is a small reduction of lesion contrast in most cases, which is also reflected in the lesion biases in Figure 10. However, as observed in Supplementary Figure 15, in case of regularized MLTR-based μ-maps, the SUV_max_ bias of the lesions is within ±10%, relative to the OSEM images using the ground truth μ-maps, in 21 out of 22 lesions. MLAA-TX-based images show lower SUV max for all lesions, compared to images using MLTR-based μ-maps. Transverse slices through lesion 11, showing the largest bias, are shown in Supplementary Figure 16, in which an increased bias in the lung can be observed in addition to reduced SUV_max_ when lutetium-based AC is used in all three scanner geometries. Figure 10 shows higher bias values and more variability in SUV_max_ of the lesions, calculated in reference to the ground truth activity maps, compared to organ biases; in which, lesion SUVmax biases in the range of −50% to +40% are observed with the 24-cm-long scanner. Visual comparison of transverse slices shown in Figures 8, 9, and Supplementary Figure 16 suggests negligible effect on lesion detectability for the two long-AFOV scanners, considering the effects from background noise.

### Weighting factor in MLAA-TX algorithm

**Figure 11:**
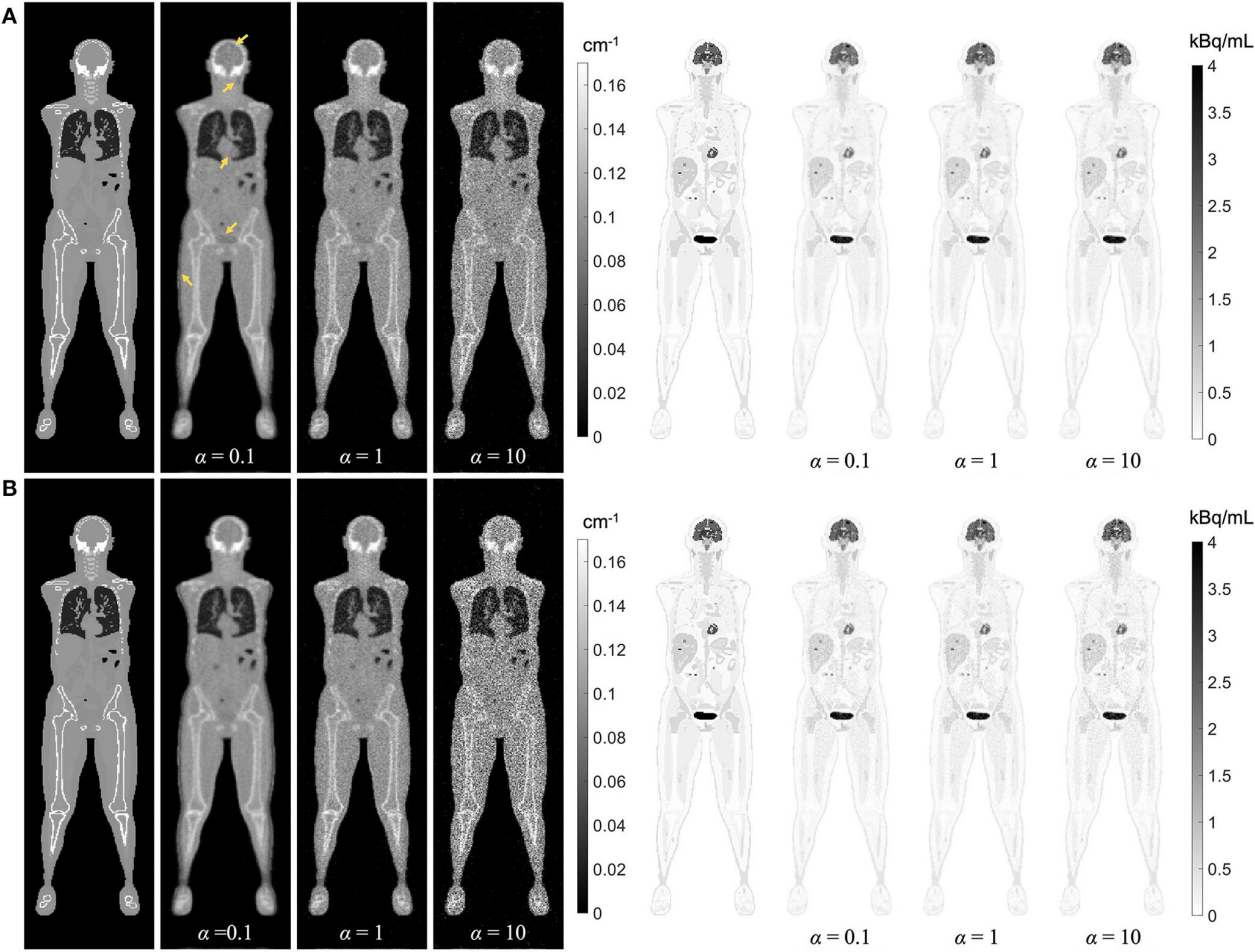
Regularized MLAA-TX reconstructions of the attenuation and activity maps with the uEXPLORER geometry using **(A)** 3 iterations and **(B)** 10 iterations, with three different weighting factors α. All reconstructions were performed using a regularization parameter β of 10,000.

Regularized MLAA-TX reconstructions of the attenuation and activity maps with the uEXPLORER geometry are compared with a weighting factor **α** of 0.1, 1, and 10, at iterations 3 and 10 in Figure 11. The transmission-emission crosstalk artifacts are reduced at higher iterations and with larger weighting factors used for the transmission scan. At iteration 3 of MLAA-TX with weighting factor 0.1, the crosstalk artifacts are the strongest and are visible in form of underestimated attenuation coefficients in all high-contrast regions (marked with yellow arrows on the μ-map). This includes the bladder, the brain, salivary glands, the myocardium, the skin, and the high-contrast lesions. Furthermore, as the weighting factor increases, the noise in the μ-maps is also increased. The crosstalk artifact and the increased noise both have impacts on the activity maps. Propagation of noise can be observed particularly in activity maps using higher weighting factors. Furthermore, larger effect of the crosstalk artifact can be observed on the activity map using **α =.1** at iteration 3, with larger biases (underestimation) in the brain, the myocardium, and the bladder. Similar effect can be also observed in SUV_max_ of the lesions in MLAA-TX images, shown in Figure 11, which show reduced SUV_max_ for all lesions compared to OSEM images using the regularized MLTR μ-maps.

### Effect of Compton scattering on lutetium-based AC

**Figure 12:**
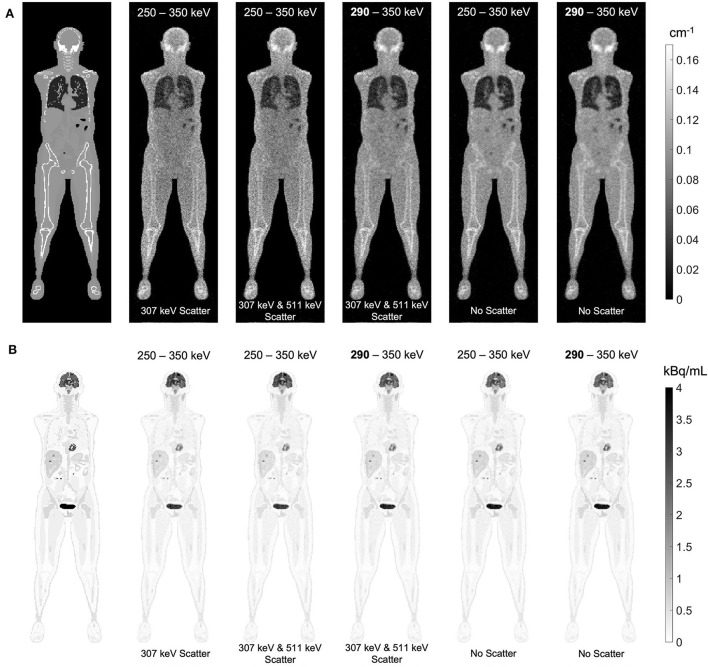
**(A)** regularized MLTR reconstructions of the μ-maps, reconstructed using two different energy windows of 250–350 keV and 290–350 keV, comparing the μ-maps with no scattered events to μ-maps that either only include the 307 keV-scattered photons or include both 307 keV and 511 keV-scattered photons. All reconstructions used a regularization parameter **β** of 10,000. **(B)** corresponding TOF-OSEM reconstructions of the activity maps using the MLTR-based μ-maps shown in the top row.

**Figure 13:**
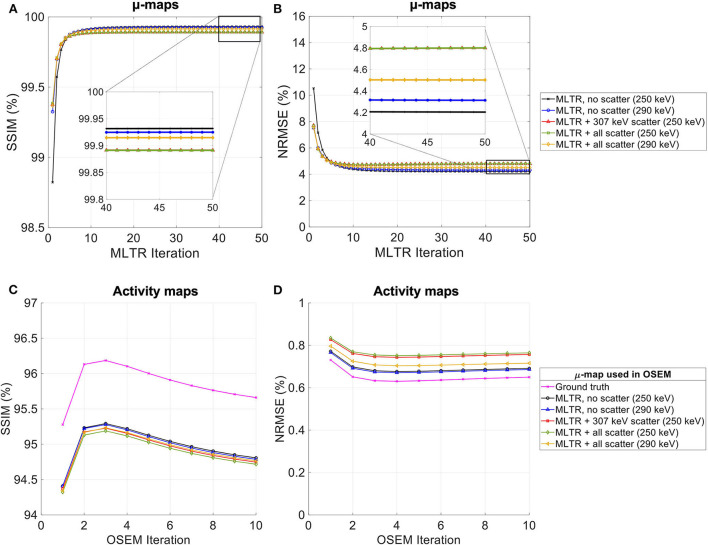
**(A,C)** SSIM and **(B,D)** NRMSE of the **(A,B)** regularized MLTR-based μ-maps and **(C,D)** activity maps attenuation corrected using the corresponding μ-maps. μ-map reconstructions were performed using two different energy windows of 250–350 keV (labeled as 250 keV) or 290–350 keV (labeled as 290 keV) and compare the μ-maps reconstructed with no scatters events (labeled as no scatter) to μ-maps that either only include the 307 keV-scattered photons (labeled as 307 keV scatter) or include both 307 keV- and 511 keV-scattered photons (labeled as all scatter). The activity images are additionally compared to OSEM reconstructions using the ground truth μ-maps, plotted in magenta color.

**Figure 14:**
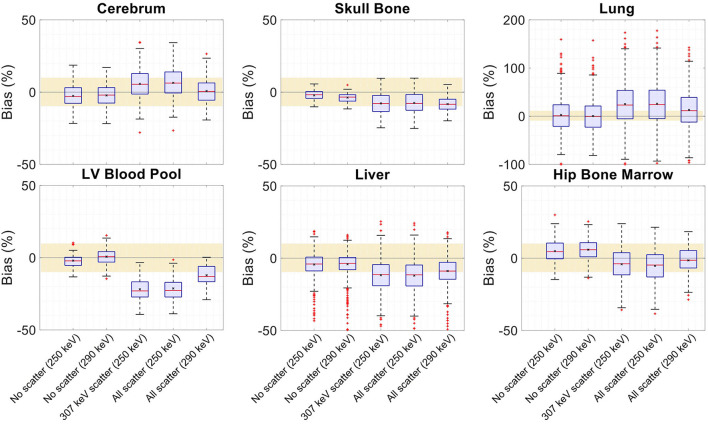
Attenuation map bias (%) distribution in selected organs of interest shown for regularized MLTR reconstructions using two different energy windows of 250–350 keV (labeled as 250 keV) or 290–350 keV (labeled as 290 keV), comparing the μ-maps reconstructed with no scatters events (labeled as no scatter) to μ-maps that either only include the 307 keV-scattered photons (labeled as 307 keV scatter) or include both 307 keV- and 511 keV-scattered photons (labeled as all scatter). The region marked with a yellow color depicts the ±10% range. Note: All box plots share the same labels. Results for the lungs are shown on a different y-axis range due to higher variability.

**Figure 15:**
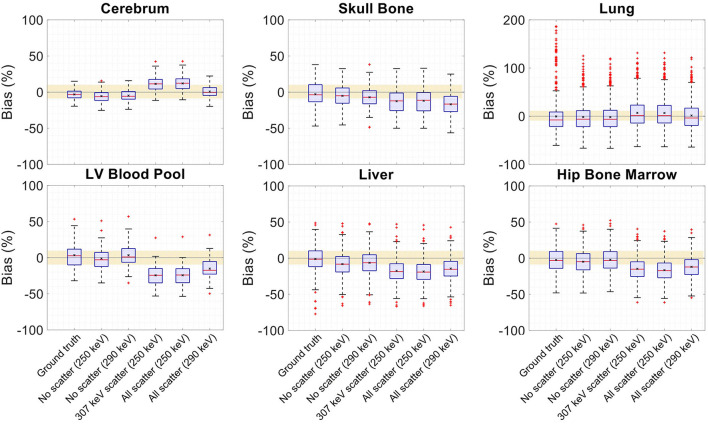
Activity map bias (%) distribution in selected organs of interest shown when OSEM reconstructions are used with regularized MLTR-based μ-maps, reconstructed using two different energy windows of 250–350 keV (labeled as 250 keV) or 290–350 keV (labeled as 290 keV), comparing the μ-maps reconstructed with no scatters events (labeled as no scatter) to μ-maps that either only include the 307 keV-scattered photons (labeled as 307 keV scatter) or include both 307 keV- and 511 keV-scattered photons (labeled as all scatter). OSEM reconstructions using the ground truth μ-maps are shown additionally as a reference. The region marked with a yellow color depicts the ±10% range. Note: All box plots share the same labels, and the labels represent the μ-maps used for AC in the OSEM algorithm. Results for the lungs are shown on a different y-axis range due to higher variability.

The effect of Compton scattering of ^176^Lu emissions and PET 511 keV emissions on lutetium-based AC is shown in Figure 12, by comparing regularized MLTR reconstructions of the μ-maps, reconstructed using two different energy windows of 250–350 keV and 290–350 keV for the second event in the coincidence window. The μ-maps with no scattered events are compared to μ-maps that either only include the 307 keV-scattered photons or include both 307 keV- and 511 keV-scattered photons. Corresponding OSEM reconstructions of the activity maps using the aforementioned MLTR-based μ-maps are also compared. The results of SSIM and NRMSE analysis on the images are shown in Figure 13, and the bias distributions for selected organs of interest are shown in Figures 14, 15 for the μ-maps and the activity maps, respectively.

Comparing the μ-maps reconstructed with the two energy windows with no scatter shows negligible effect from increasing the energy threshold to 290 keV on image quality and quantification. SSIM and NRMSE of the μ-map using the 290 keV threshold are slightly degraded compared to the one using the 250 keV threshold; however, SSIM and NRMSE of the activity maps using these two μ-maps show negligible differences. This is also confirmed by comparison of the bias distributions, where increasing the energy threshold to 290 keV shows negligible effect on the μ-map and activity map biases, and bias in all organs of interest remains within ±10%.

Comparing the μ-maps that only include the 307 keV scatter to the μ-maps that include both 307 keV and 511 keV scatter shows negligible differences in SSIM, NRMSE, and bias distribution in both μ-maps and their corresponding activity maps. However, although the μ-maps including the scatter were scaled, they still suffer from scatter artifacts, particularly in the abdominal region. This results in degradation of SSIM, NRMSE, and bias in both μ-maps and activity maps, when compared to μ-maps with no scatter. In all organs of interest, except for the lungs, the activity map mean bias exceeds ±10%, when scattered events are included in the μ-maps. The left ventricle blood pool, the liver, and the hip bone marrow show the highest mean biases in the activity maps, with 24%, 18%, and 17% bias, respectively, when the scatter is included in the μ-maps.

Increasing the energy threshold to 290 keV improves the image quality and quantification of the μ-maps when the scatter is included. Comparing the SSIM and NRMSE plots for the μ-maps and their corresponding activity maps shows that using a 290 keV threshold reduces the difference between the μ-maps reconstructed with and without the scatter. This is also observed in the bias distribution plots for attenuation and activity maps, where bias is reduced in all organs of interest, except for the skull bone, when the 290 keV threshold is used in the μ-maps that include the scatter. However, the activity map mean bias still exceeds the ±10% range in most organs of interest, except for lungs and the cerebrum. In this case, the left ventricle blood pool, the liver, and the hip bone marrow biases are reduced to 15%, 14%, and 12%, respectively.

## Discussion

The simulated uEXPLORER geometry shows an overestimation of the sensitivity, compared to the measured total sensitivity and peak sensitivity values reported by Spencer et al. (11). This is probably due to absence of count loss and detector efficiency modeling in the GATE model used. However, this overestimation is included for all three scanner geometries, and the presented comparisons should be minimally impacted by it. The shape of the axial sensitivity profile of the simulated uEXPLORER geometry matches the measurement results with a 170-cm line source and includes the triangular shapes introduced by the maximum unit difference policy. The comparison of the sensitivity profile to the other two shorter geometries is only representative of the scatter-free sensitivity at 511 keV, and the sensitivity differences between the three geometries become smaller as the attenuation medium is introduced and for 307 keV emissions. Furthermore, the 170-cm line source sensitivity, representing a standard human height, has emissions outside the scan length of the two shorter scanner geometries, which also affect the sensitivity profile measurements. The simulated line source and the XCAT phantom were both centered in the AFOV of the uEXPLORER scanner, similar to the scanning protocols performed on the clinical system. For the two shorter scanner geometries, the scan positions for the simulations were selected to cover the head to thighs. Depending on the clinical application, the scan position can be adjusted for the two long-AFOV scanner geometries differently to move the brain and the chest region to higher sensitivity areas.

Comparison of the reconstructed images of the simulated NEMA image quality phantom and its contrast recovery coefficient and background variability to the results presented by Spencer et al. (11) using 2.344-mm voxels and no PSF modeling shows good agreement between the results obtained with the commercial scanner compared to the simulated model and the reconstruction framework used in this work. This suggests that similar image quality can be expected between experimental implementation of the methodology used in this paper and the simulated results upon successful incorporation of data corrections.

Comparing the effect of the acceptance angle and scan duration on quantification of the lutetium-based μ-maps shows larger effects from reducing the scan duration. This suggests that the long-AFOV scanners will mainly benefit from the duration of a single-bed-position acquisition of the transmission data that can be acquired simultaneously with the emission data. The smaller gain observed in sensitivity at acceptance angles higher than 32° can be explained by increased Compton scattering probability at lower energies and with more oblique LORs, leading to higher attenuation. This also suggests that future implementations of the μ-map reconstruction algorithm can use a restricted acceptance angle of 32° to speed up the reconstructions. The small absolute changes of SSIM and NRMSE observed after convergence for both reduced acceptance angles and reduced scan times in all cases are expected due to the effects from regularization in controlling the noise and the contribution of the voxel values that are insignificantly affected during the reconstruction. However, the additional regional bias analysis performed on the images shows that SSIM and NRMSE alone are not sufficient figures of merit to assess the quantification effects.

Three points of consideration are required to interpret the presented bias distribution comparison plots and to understand why they have been used instead of the commonly used bar plots of mean biases. Firstly, since the regularization parameter was tuned for each scan to optimize SSIM and NRMSE, this does not translate into optimized bias distribution in the images. Therefore, as a reference, non-regularized reconstructions were compared in all cases to ensure that the conclusions about bias comparisons are valid. This effect can be observed in the bias distribution plots using regularized images, where lower statistic images in some cases show smaller variability (represented by the whiskers) due to larger effects from regularization. However, in non-regularized bias distributions, whiskers correlate with image noise and count statistics. Secondly, in a similar concept, in the comparisons of the activity maps for the three scanner geometries, since the iteration number (and the subset number) was chosen for each scanner to optimize the SSIM and NRMSE, one-to-one comparison of the organ bias values was affected even when non-regularized μ-map reconstructions were used for AC. This particularly can make the comparison between the uEXPLORER and the one-meter-long scanner difficult, as the difference between the two scanners becomes smaller and the lower-sensitivity scanner uses lower number of iterations with less noise amplification. Finally, in low-count situations, such as the low-dose PET scan simulated in this study, the non-negativity constraint in the OSEM algorithm might result in mean biases with absolute values closer to zero, when the bias distribution in the organ of interest is negative and increased noise in the image pushes the mean value closer to zero. An example of this can be observed in Supplementary Figure 13 for hip bone marrow bias in the 24-cm-long scanner, where using the ground truth μ-map leads to a larger absolute mean bias, whereas using the non-regularized MLTR-based μ-map is introducing more noise into the activity map and artificially pushes the mean bias toward zero by the non-negativity constraint.

In this work, bias of the activity map is always calculated in reference to the known simulated ground truth activity map. This is in contrast to previous studies using measurement data, in which activity map bias is calculated in reference to the activity maps using CT-based AC. As shown in this work, there is a baseline bias in the activity maps when ground truth μ-maps are used for AC, which are caused by partial volume effect, spill-over from neighboring voxels, feature size, and contrast, and, in general, is also affected by OSEM convergence. While, in human scans, such ground truth activity maps do not exist, the biases calculated relative to CT-based AC are expected to be smaller than the biases relative to the ground truth, as shown in this paper. Given that a baseline bias exists with ground truth AC, the bias distributions have to be compared, considering their baseline values. An example of such effect, which may appear counter-intuitive at the first glance, is the skull bone activity bias in the 24-cm-long scanner shown in Supplementary Figure 13, where using the lutetium-based μ-maps leads to lower bias in the activity maps, compared to using the ground truth μ-maps. This can be explained by the skull bone bias in the μ-maps presented in Supplementary Figure 12, where using lutetium-based μ-maps leads to underestimation of the attenuation coefficients, consequently resulting in a reduction of the corresponding voxel values in the activity map. Given that the skull bone region in the activity map is already suffering from a positive bias with the ground truth μ-map, probably due to the spillover from brain activity, the relative reduction translates into a mean bias closer to the zero. A similar effect can also be observed in Figure 15, in which the OSEM image using the ground truth μ-map shows more outliers in the lung region compared to lutetium-based AC images, while showing a smaller interquartile range. This can be explained by the existing nonuniform structures in the lung due to presence of lung bronchi, which are better recovered when ground truth μ-maps are used as observed in reconstructed images shown in Figure 9. Lastly, it has to be noted that the activity map bias variability observed within different organs of interest is also affected by the count statistics in each region, with larger variability in low-statistic regions compared to a high-statistic region such as the cerebrum.

Current investigation of the effects of using lutetium-based AC on lesion quantification suggests that negligible impact on lesion detection can be expected when a proper regularization parameter is used; however, SUV_max_ of the lesions can be affected even in long-AFOV scanners, particularly with MLAA-TX reconstruction, with up to 17% bias (underestimation) relative to OSEM images using the ground truth μ-maps. Large variability and large absolute bias values observed in Figure 10 are expected to be due to partial volume effects, in addition to the effects from OSEM convergence and high statistical noise present in the simulated ultralow-dose scan. As convergence speed of the OSEM algorithm is affected by object size, slower convergence can be expected for the lesions compared to larger organs. Therefore, the OSEM iteration number selected for each scanner geometry may not provide the optimized lesion bias, while trying to maintain an acceptable image noise level. This has a larger effect on the 24-cm scanner results, in which a lower number of subsets and iterations was used due to lower sensitivity of the scanner. As a result, comparison of lesion SUV_max_ bias in Figure 10 shows unexpected results for a number of lesions, such as lesions 9 and 21, in which the 24-cm long shows the smallest absolute bias compared to two longer-AFOV scanners. Therefore, to provide a better comparison of the lesion quantification among three scanner geometries and to demonstrate the effect from convergence, lesion SUV_max_ bias was plotted as a function of equivalent MLEM iterations (OSEM iterations × number of subsets) for all 22 lesions, shown in Supplementary Figure 17. While reconstructions of the lesions with the 24-cm-long scanner are far from convergence at OSEM iteration 2 (equivalent MLEM iteration 8), they are approaching convergence for the one-meter and the uEXPLORER geometry at iteration 2 (equivalent MLEM iteration 20) and iteration 3 (equivalent MLEM iteration 30), respectively. Furthermore, upon availability of sufficient counts and in a lower-concentration background, the lesion SUV_max_ values are expected to be underestimated at convergence due to partial volume effects. However, when limited counts are available, large variations of SUV_max_ bias can be expected in different replicates of the data, among which positive biases can also be observed. This is the case for the simulated ultralow-dose scan, particularly with the low sensitivity of the 24-cm scanner, where large positive biases are observed; and this similarly applies to the comparisons of the closely obtained results for the two larger-AFOV scanners. Although in most lesion detection tasks in oncology, there are less concerns about diagnostic imaging radiation dose, and availability of high-quality CT correlation is preferred, the lesion quantification evaluation in this study may be relevant for applications where several longitudinal scans are involved and a high-quality CT image may not be required or possible every time. The lesions in this case could represent any small focus of uptake that could occur in various disease conditions using different tracers, where ultralow-dose PET scans are of interest. Examples of such cases are quantification of foci of inflammation in joints in ^18^F-FDG arthritis studies or quantification uptake in lymph nodes at several time points in immunological studies with ^89^Zr-labeled tracers. Further investigation is required to compare the lesion quantification accuracy in such cases, compared to AC with ultralow-dose CT scans. Potential improvements can be expected by combining the information from lutetium transmission data with other *a priori* knowledge from ultralow-dose CT scans, scout scans, or previous CT scans of the patients using kernel-based reconstruction algorithms and deep-learning-based methods.

While, in this study, MLAA-TX has shown no significant advantage over MLTR-based AC, it has to be noted that, at higher-count PET scans, the PET data can contribute more to estimation of the μ-map, as shown in the previous studies (34, 40). However, as depicted in Figure 11, the TOF-MLAA algorithm may require more iterations to converge to a solution that does not suffer from transmission-emission crosstalk artifacts. The lower noise observed in the MLAA-TX μ-maps compared to the MLTR μ-maps could be due to availability of attenuation information in the TOF-PET data.

Comparison of the μ-maps with and without contamination from Compton-scattered 511 keV photons has shown that the main component contributing to AC inaccuracy is the Compton scattering of 307 keV photons; and the 511 keV scatter is successfully removed by the TOF discrimination criteria. While the current study only evaluated ultralow-dose PET scans, it has to be noted that, without the TOF criteria, which is applied to the transmission coincidences to differentiate the scattered emission coincidences from transmission coincidences, the μ-maps were heavily contaminated by the scattered emission data, even with such a low-count PET scan, and obtaining μ-maps in the abdomen and pelvis region was not possible. This suggests that scatter contamination from emission data can be successfully removed by the TOF criteria to a large extent. However, the effectivity of the TOF criteria needs further evaluation for higher dose PET scans, non-pure positron emitter tracers, different subject sizes, and different system TOF resolutions.

Increasing the lower energy threshold from 250 keV to 290 keV offers several advantages and has shown to have a negligible effect on AC accuracy. As shown in Supplementary Figure 18, increasing the energy threshold to 290 keV removes only 26% of the 307 keV photons that did not scatter, but removes 67% of the 307 keV-scattered photons. This leads to a lower scatter fraction in the transmission data; as a result of which, the initial reconstruction of the μ-map provides a better estimate of the scatter sinogram and requires less iterations during 307 keV SC. Furthermore, increasing the energy threshold reduces both the singles and coincidence data rates, which can be particularly important for implementing this method on a clinical scanner. However, although the main application of this work is expected to be in ultralow-dose PET scans, experimental validation of current results is still required at different PET count-rate levels, since increased pileup effect can be expected at high count rates.

The current study was limited to simulation data and, as a first step in future studies, the quantification errors due to SC need to be evaluated. The results presented in this study did not include the effects from errors in the data correction chain. Particularly, simplifications made in removing the random and scattered coincidence are expected to underestimate the noise. Therefore, the reconstructed images including SC and random correction are expected to suffer from more noise in similar experimental settings. Moreover, a number of challenges arise when experimental data are used that require further investigation. These include the effects from detector calibration, dead-time calibration, and sensitivity of MLAA algorithm to TOF calibration and normalization errors. Another technical challenge in acquiring the transmission data in total-body PET would be the increased file size of the list-mode data and possible count rate limitations in readout electronics, as the system is operated with a lower energy threshold and wider coincidence time window. Furthermore, while the current study suggests that MLTR reconstructions of the transmission data are sufficient for accurate AC in total-body PET and they are less computationally expensive than MLAA or MLAA-TX algorithms, use of mashing can significantly reduce the reconstruction time in this case, especially when additional computational time may be needed for 307 keV SC. Future work will also study the effect of mashing on AC accuracy. Finally, deep-learning-based approaches can be incorporated into this framework to potentially improve the quantification accuracy and computational time of the lutetium-based AC in total-body PET.

## Conclusion

This simulation study has demonstrated successful use of lutetium transmission data for AC of ultralow-dose PET scans in total-body PET scanners. Quantification of PET activity and μ-maps was minimally affected in whole-body 20-min scan simulations of a 3D XCAT phantom, and mean bias in all analyzed organs of interest was within a ±10% range in reference to the ground truth. The two simulated long-AFOV total-body PET scanners (i.e., the uEXPLORER using a single bed position and a one-meter-long scanner using two bed positions) showed comparable performance, quantitatively superior to the results obtained with an 8-bed-position scan on a conventional 24-cm-long scanner. With lutetium-based AC, SUV_max_ bias was within ±10%, relative to OSEM reconstructions using ground truth μ-maps, for 21 out of 22 lesions and reached −17% for an 8-mm lesion in the lung. MLAA-TX reconstructions of the simulated ultralow-dose PET scan suggest that MLTR reconstructions of the μ-maps may be sufficient for accurate AC in low-count total-body PET data. Furthermore, quantification accuracy of the μ-maps can be affected in certain organs of interest, with increased biases outside the ±10% range, when scan duration is reduced to 5 min and acceptance angles of 17° or less are used. Finally, the quantification analysis performed with the μ-maps including the Compton-scattered data suggests that implementation of an SC method for the 307 keV photons will be required and an increased energy threshold of 290 keV can be used to reduce the computational costs and data rates, with negligible effects on PET quantification. In conclusion, this work has provided a groundwork for clinical implementation of lutetium-based AC in total-body PET. We believe that the utilization of this method has greatest implications for ultralow-dose PET scans that are now made possible with total-body PET scanners; and transferring these methods into clinical and research practice will enable wider applications of total-body PET, such as ultralow-dose pediatric imaging or multiple longitudinal scans in healthy subjects, leading to better understanding of human health.

## Data availability statement

The raw data supporting the conclusions of this article will be made available by the authors upon request, without undue reservation.

## Ethics statement

Ethical review and approval was not required for this study in accordance with the local legislation and institutional requirements.

## Author contributions

NO: design, methodology, investigation, analysis, and writing of the initial manuscript. LC, EL, and YA: methodology. RB, TM, JQ, and SC: methodology and supervision. All authors contributed to the article and approved the submitted version.

## Funding

Funding for this work was provided by NIH grants R35 CA197608, R01CA206187, which is supported by NCI, NIBIB, and the Office of the Director, and R03 EB032457.

## Conflict of interest

UC Davis has a research agreement and a sales-based revenue-sharing agreement with United Imaging Healthcare. RB and SC are principal investigators on a research grant funded by United Imaging Healthcare. EL is a full-time employee at UIH America, Inc. The remaining authors declare that the research was conducted in the absence of any other commercial or financial relationships that could be construed as a potential conflict of interest.

## Publisher's note

All claims expressed in this article are solely those of the authors and do not necessarily represent those of their affiliated organizations, or those of the publisher, the editors and the reviewers. Any product that may be evaluated in this article, or claim that may be made by its manufacturer, is not guaranteed or endorsed by the publisher.
