# Supplementary material for: Lutetium background radiation in total-body PET—A simulation study on opportunities and challenges in PET attenuation correction
Source: Front Nucl Med. 2022 Aug 10;2:963067. doi: 10.3389/fnume.2022.963067 (PMC9513593; doi:10.3389/fnume.2022.963067)
Supplement: Supplementary file 1 [file Data_Sheet_1.docx]

# Supplementary Material

| Total number of crystals | 564,480 |
| --- | --- |
| Crystal size | 2.76 × 2.76 × 18.1 mm^3^ |
| Crystal pitch | 2.85 mm |
| Crystal material | Ce:LYSO (Lu_1.8_Y_0.2_SiO_5_) |
| Crystal density | 7.11 g cm^-3^ |
| Crystal mass fractions | Lutetium = 71.447%  Yttrium = 4.034%  Silicon = 6.371%  Oxygen = 18.148% |
| Number of transaxial detector modules | 24 |
| Number of detector blocks per transaxial module | 5 |
| Number of crystals per transaxial detector block | 7 |
| Transaxial detector block pitch | 20.03 |
| Crystal ring diameter | 786 mm |
| Number of axial PET units | 8 |
| Number of axial detector blocks per PET unit | 14 |
| Number of crystals per axial detector block | 6 |
| Axial detector block pitch | 17.17 mm |
| Axial PET unit pitch | 242.84 mm |
| Total axial length | 1940.2 mm |

Supplementary Table 1. Design specifications of the uEXPLORER scanner

Supplementary Table 2. Size and contrast specifications of the 22 lesions in the XCAT phantom. The 8-mm diameter lesions contained 6 voxels and 12-mm diameter lesions contained 12 voxels in the 4-mm isotropic voxel grid, both containing 4 voxels in their central slice.

| Lesion index | Lesion location | Lesion-to-background ratio | Lesion diameter (mm) |
| --- | --- | --- | --- |
| 1 | Liver | 6 | 8 |
| 2 | Liver | 4 | 12 |
| 3 | Liver | 2.4 | 8 |
| 4 | Liver | 8 | 8 |
| 5 | Liver | 2.4 | 12 |
| 6 | Left lung | 12 | 12 |
| 7 | Left lung | 12 | 8 |
| 8 | Left lung | 8 | 8 |
| 9 | Right lung | 12 | 12 |
| 10 | Right lung | 12 | 8 |
| 11 | Right lung | 8 | 8 |
| 12 | Pelvis | 10 | 12 |
| 13 | Pelvis | 8 | 8 |
| 14 | Pelvis | 12 | 12 |
| 15 | Pelvis | 8 | 8 |
| 16 | Neck | 4.6 | 8 |
| 17 | Neck | 4.6 | 8 |
| 18 | Neck | 24 | 12 |
| 19 | Neck | 16 | 12 |
| 20 | Brain | 2.8 | 8 |
| 21 | Brain | 2.6 | 12 |
| 22 | Brain | 3 | 12 |

Supplementary Table 3. Spherical VOI specifications used for the bias-variance analysis.

| VOI location | Number of VOIs | VOI diameter (mm) | Mean ground truth activity concentration (Bq/mL) |
| --- | --- | --- | --- |
| Cerebrum | 6 | 15 | 2400 |
| Skull bone | 2 | 15 | 196 |
| Liver | 4 | 30 | 693 |
| Lung | 4 | 30 | 174 |
| Left ventricle blood pool | 1 | 20 | 615 |
| Hip bone marrow | 6 | 20 | 600 |


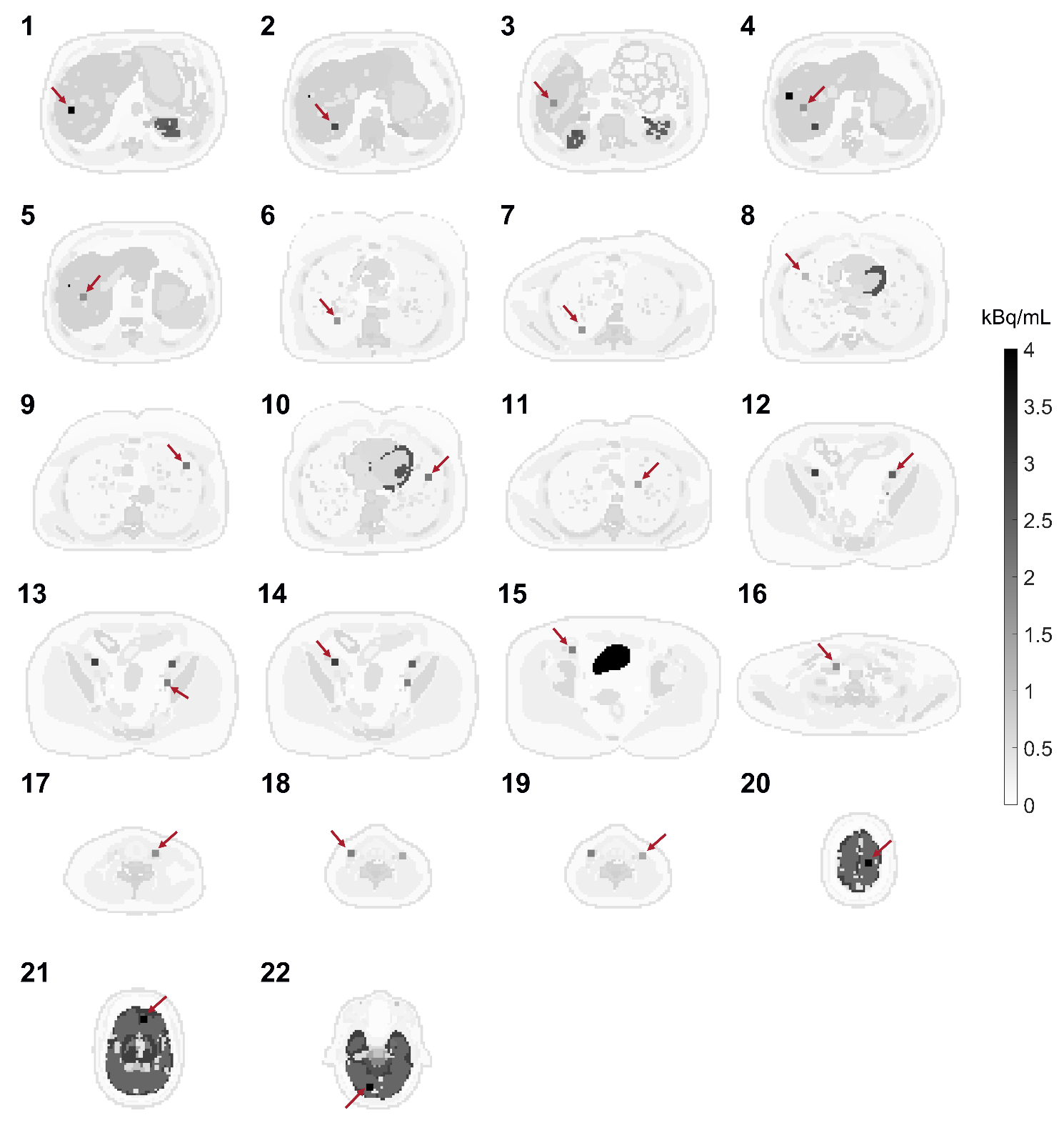


Supplementary Figure 1. Transverse slices through the centers of the 22 lesions of the XCAT phantom are shown, with red arrows pointing to the location of each lesion.

**
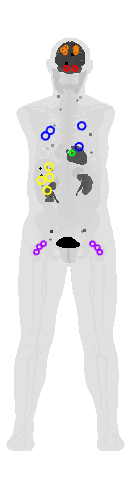
**
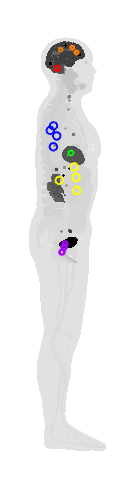


Supplementary Figure 2. Position of the 23 VOIs placed on images for bias-variance evaluation show on maximum intensity projection image of the ground truth activity map on A) coronal and B) sagittal views (orange: cerebrum, red: skull bone, yellow: liver, blue: lungs, green: left ventricle blood pool, purple: hip bone marrow).


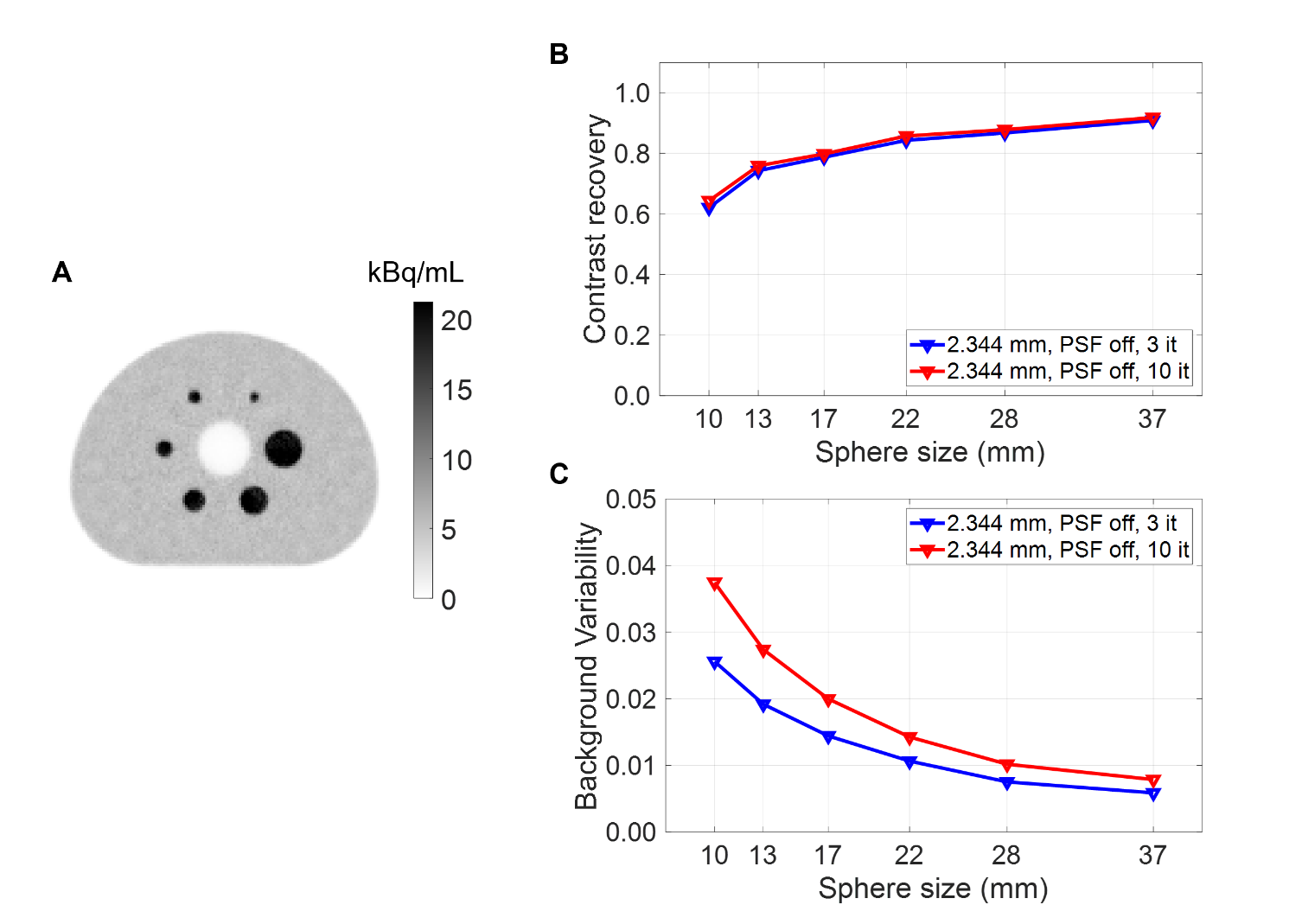


Supplementary Figure 3. A) Reconstructed image of the simulated 20-min scan of the NEMA NU 2 image quality phantom using 2.344-mm isotropic voxels, 3 iterations (10 subsets), with no PSF modelling; and the results of NEMA analysis, including B) contrast recovery coefficient and C) background variability performed on images from iteration 3 and 10.


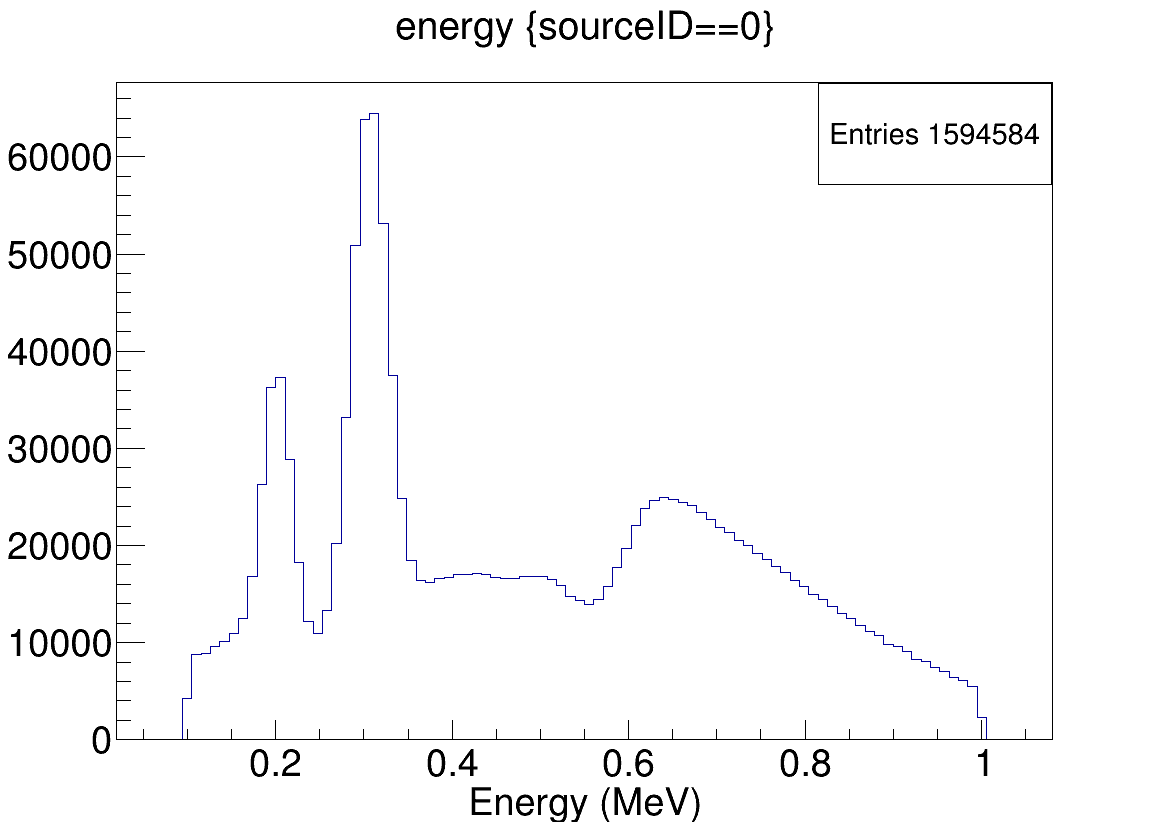

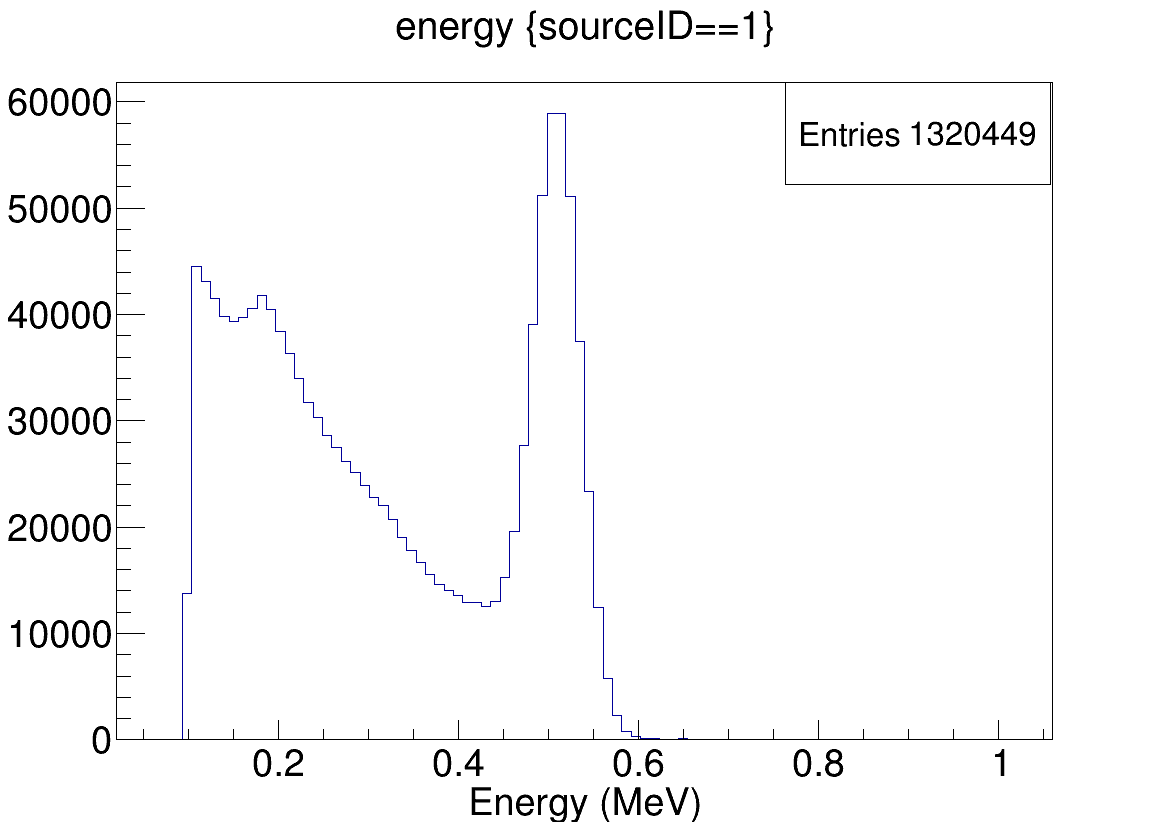

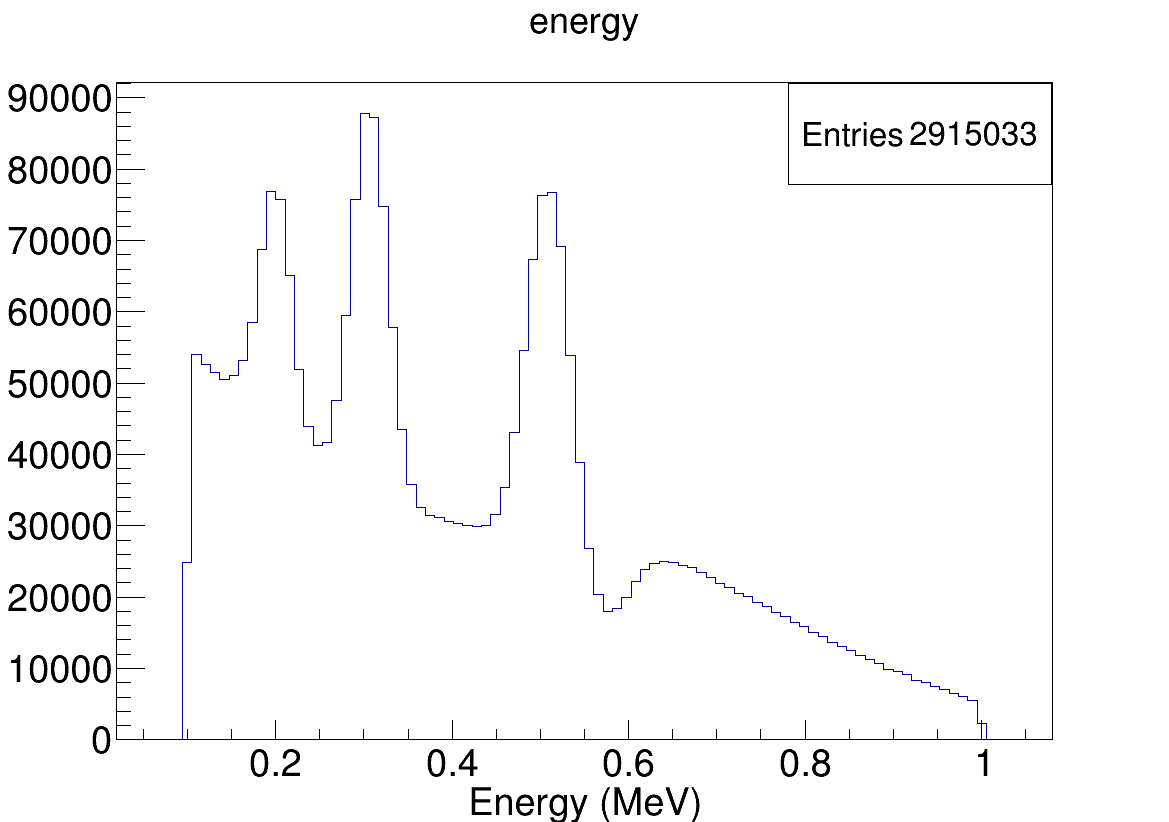


A

B

C

Supplementary Figure 4. Singles energy spectrum of a 50-ms simulation frame of the XCAT phantom in the uEXPLORER geometry, showing the contributions from A) the lutetium background radiation emissions, B) ^18^F emissions in the XCAT phantom, and C) all emissions together.

**
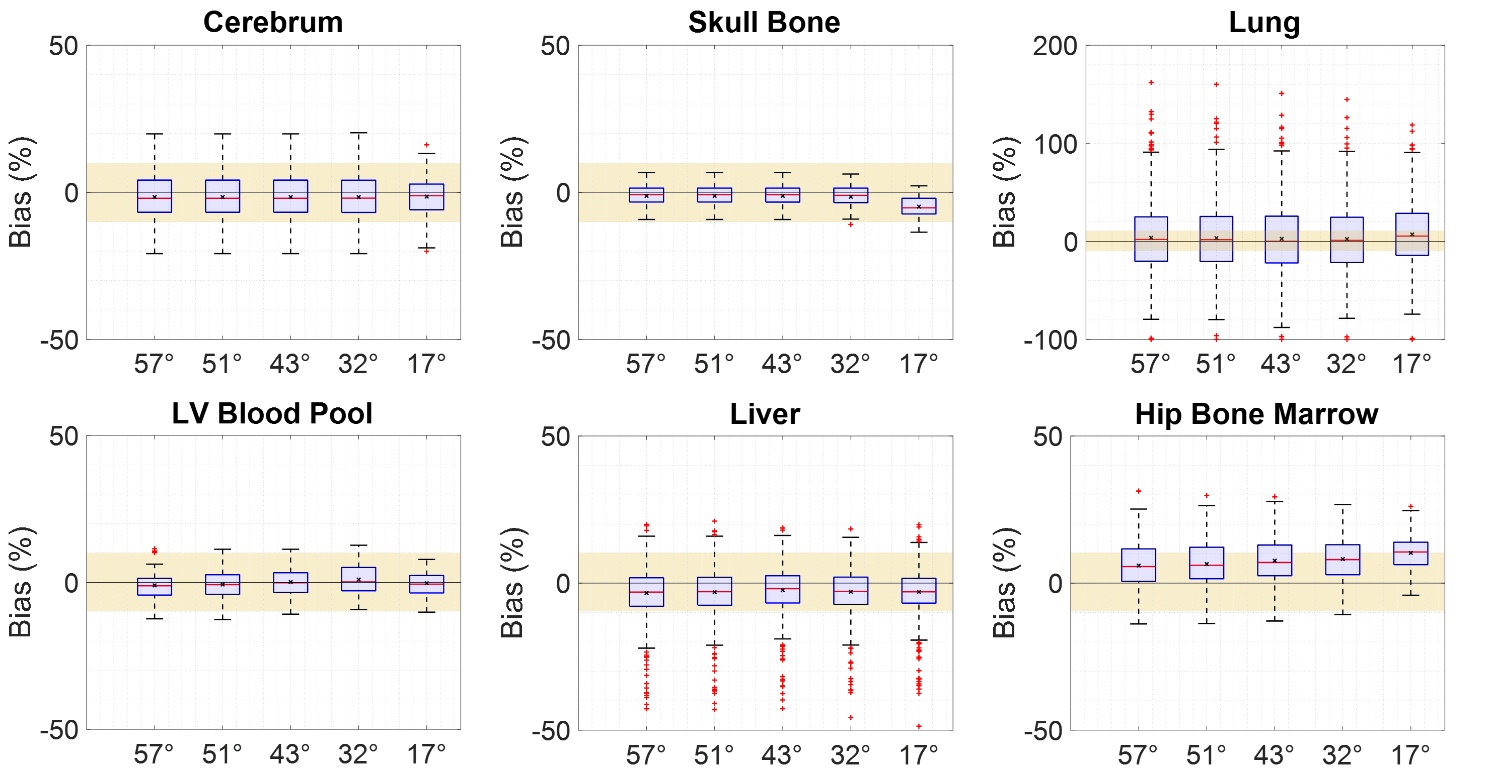
**

Supplementary Figure 5. Bias (%) distribution in selected organs of interest calculated from MLTR reconstructions of the μ-map with regularization, using the uEXPLORER geometry with different acceptance angles. The region marked with a yellow color depicts the ±10% range. Note: Results for the lungs are shown on a different y-axis range due to higher variability.


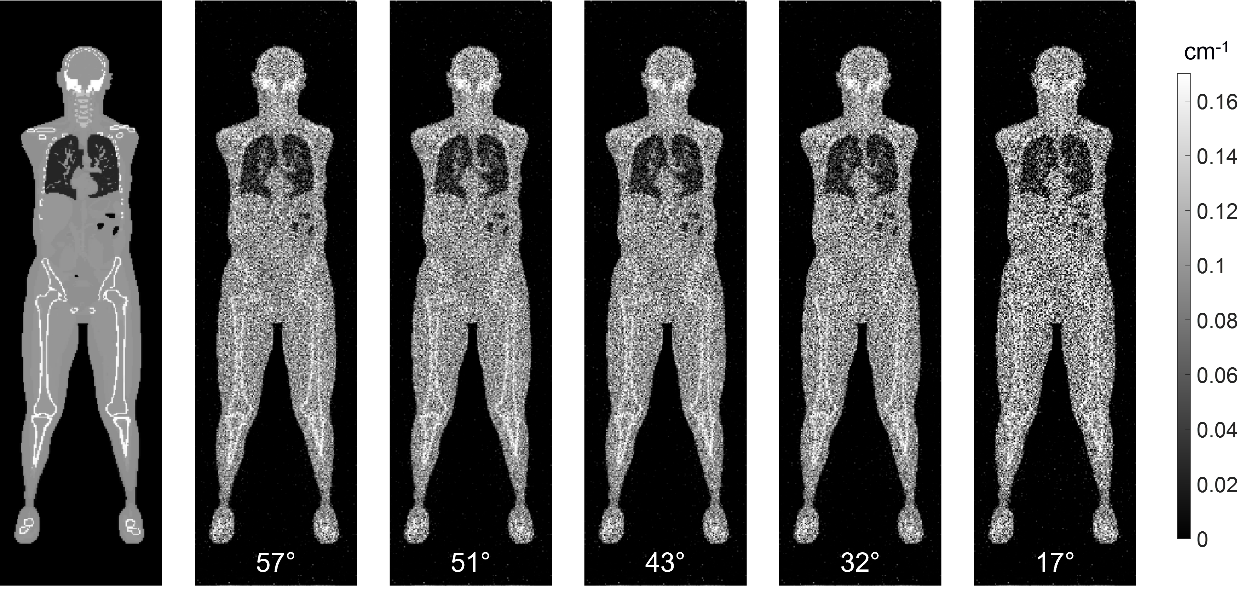


Supplementary Figure 6. Selected coronal slices from MLTR reconstructions of a 20-min lutetium transmission scan on the uEXPLORER, using maximum acceptance angles ranging from 57° to 17°, compared to the ground truth simulated μ-map. No regularization was used for the reconstructions.


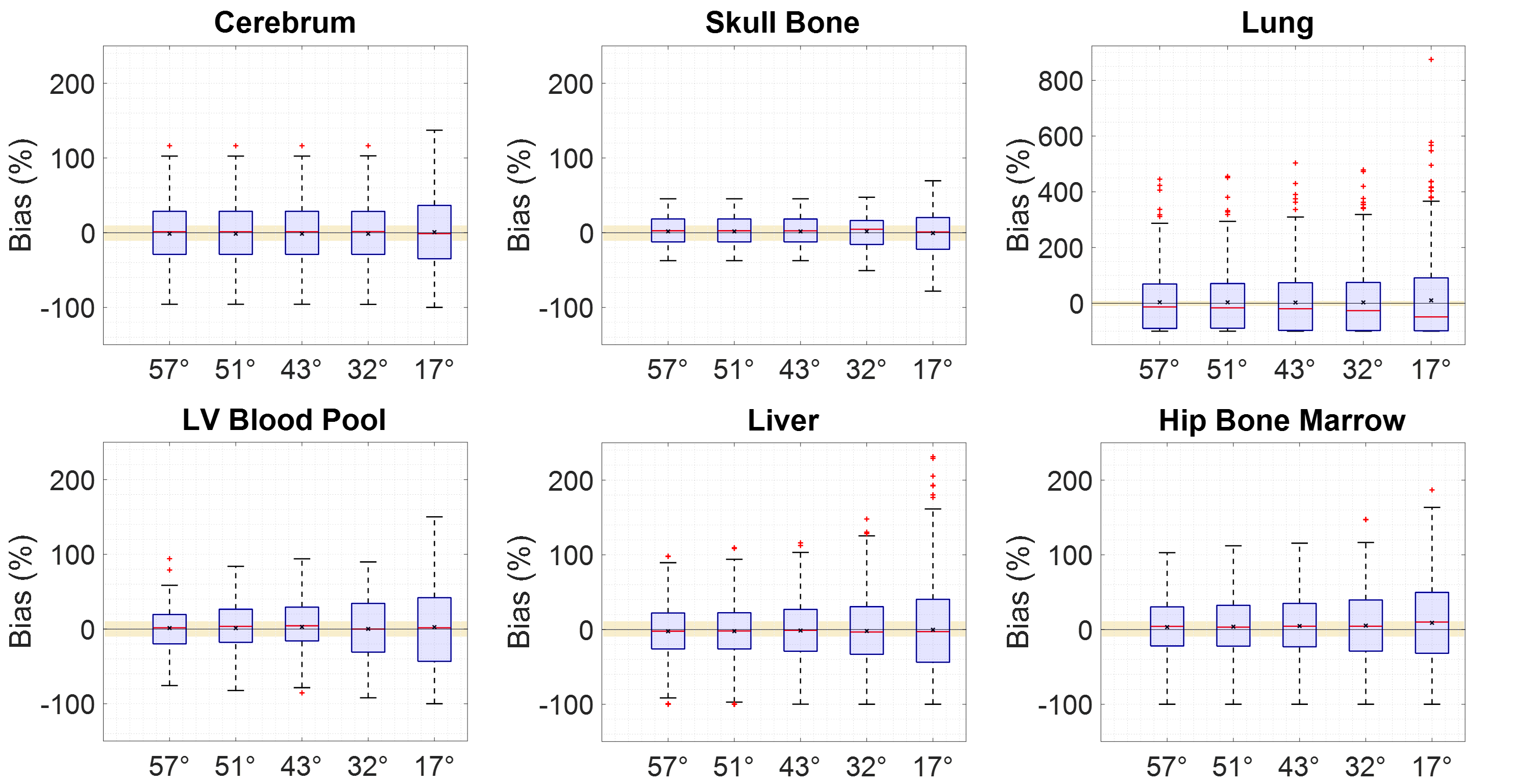


Supplementary Figure 7. Bias (%) distribution in selected organs of interest calculated from MLTR reconstructions of the μ-map with no regularization, using the uEXPLORER geometry with different acceptance angles. The region marked with a yellow color depicts the ±10% range. Note: Results for the lungs are shown on a different y-axis range due to higher variability.


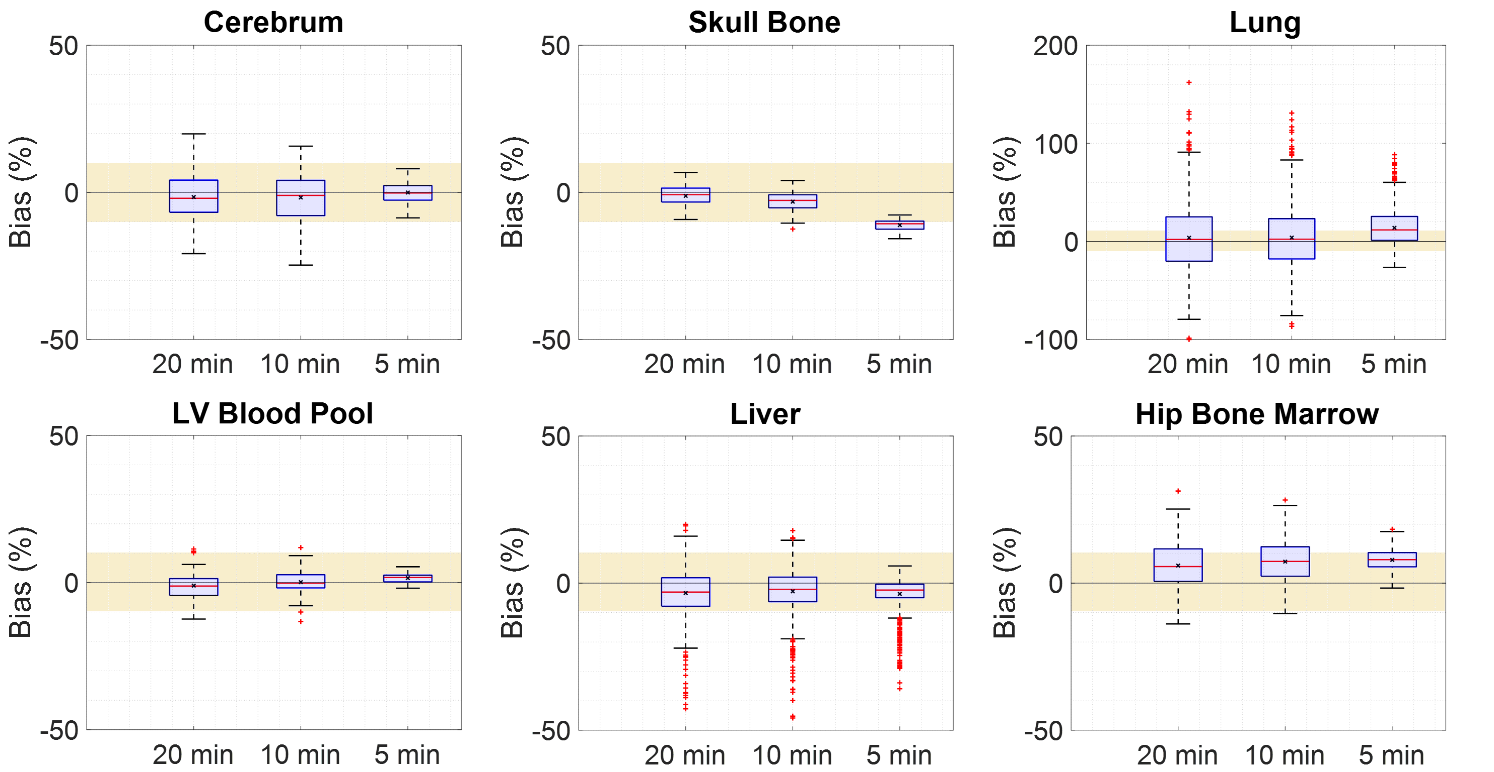


Supplementary Figure 8. Bias (%) distribution in selected organs of interest calculated from MLTR reconstructions of the μ-map with regularization, using the uEXPLORER geometry with different scan durations of 20 min, 10 min, and 5 min. The region marked with a yellow color depicts the ±10% range. Note: Results for the lungs are shown on a different y-axis range due to higher variability.


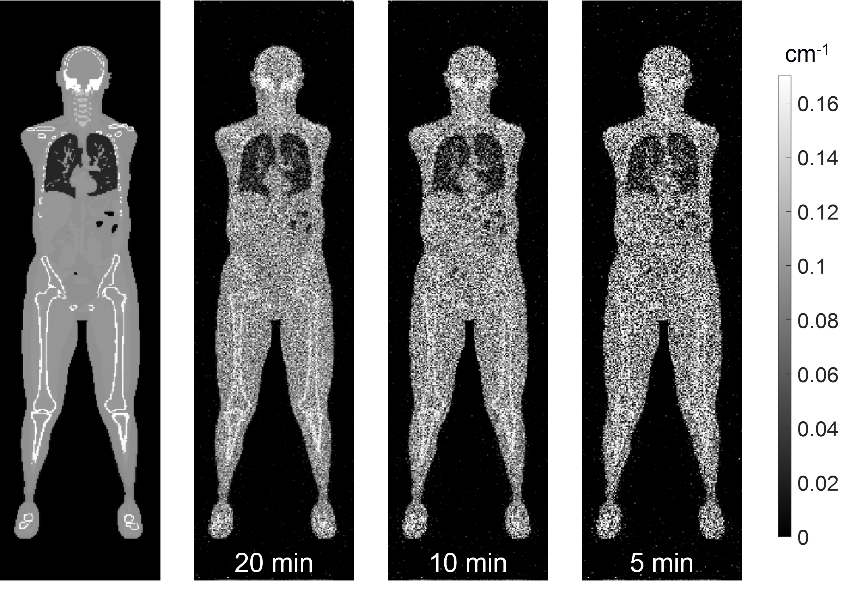


Supplementary Figure 9. Selected coronal slices from MLTR reconstructions of a lutetium transmission scan on the uEXPLORER, with scan durations of 5 min, 10 min, and 20 min, compared to the ground truth simulated μ-map. No regularization was used for the reconstructions.


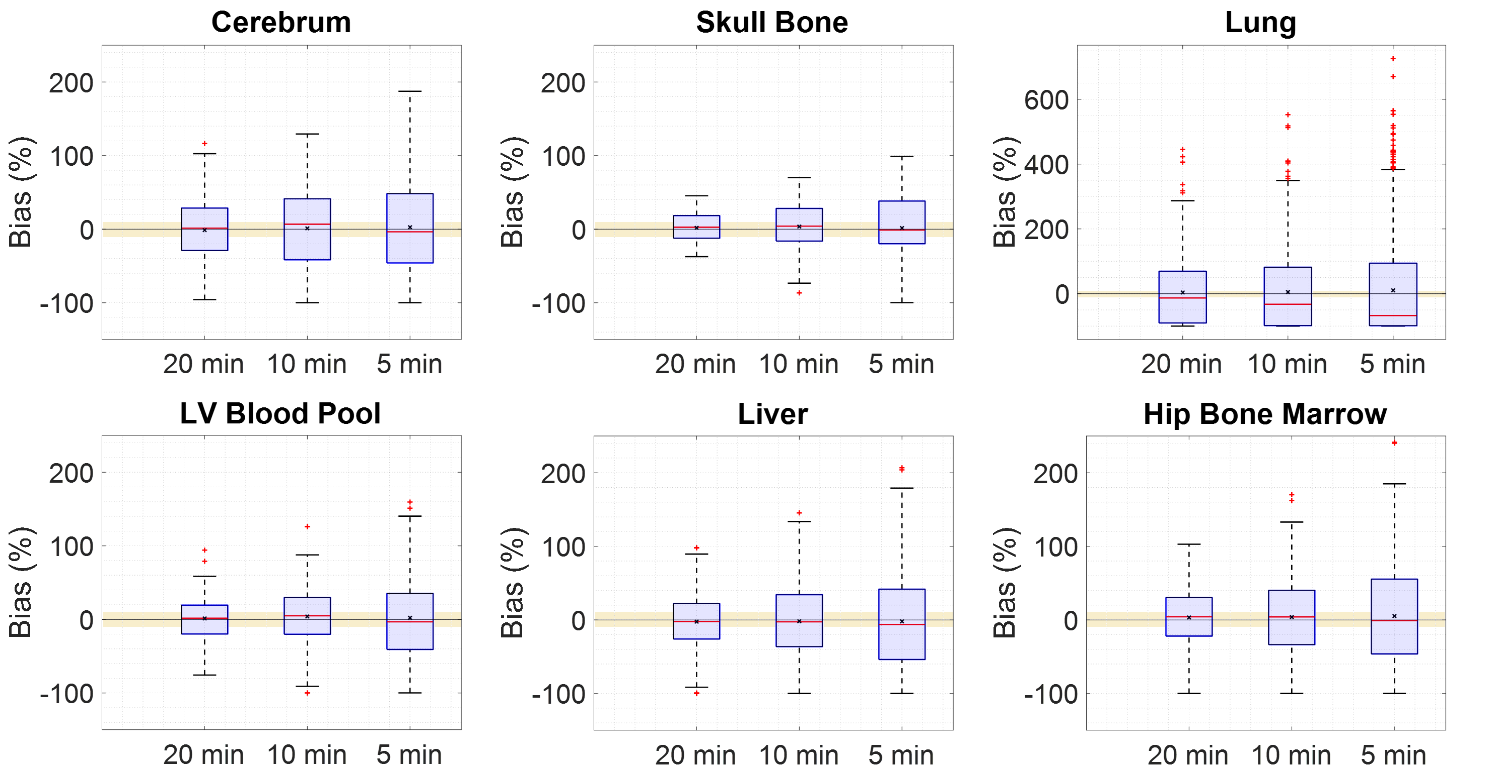


Supplementary Figure 10. Bias (%) distribution in selected organs of interest calculated from MLTR reconstructions of the μ-map with no regularization, using the uEXPLORER geometry with different scan durations of 20 min, 10 min, and 5 min. The region marked with a yellow color depicts the ±10% range. Note: Results for the lungs are shown on a different y-axis range due to higher variability.


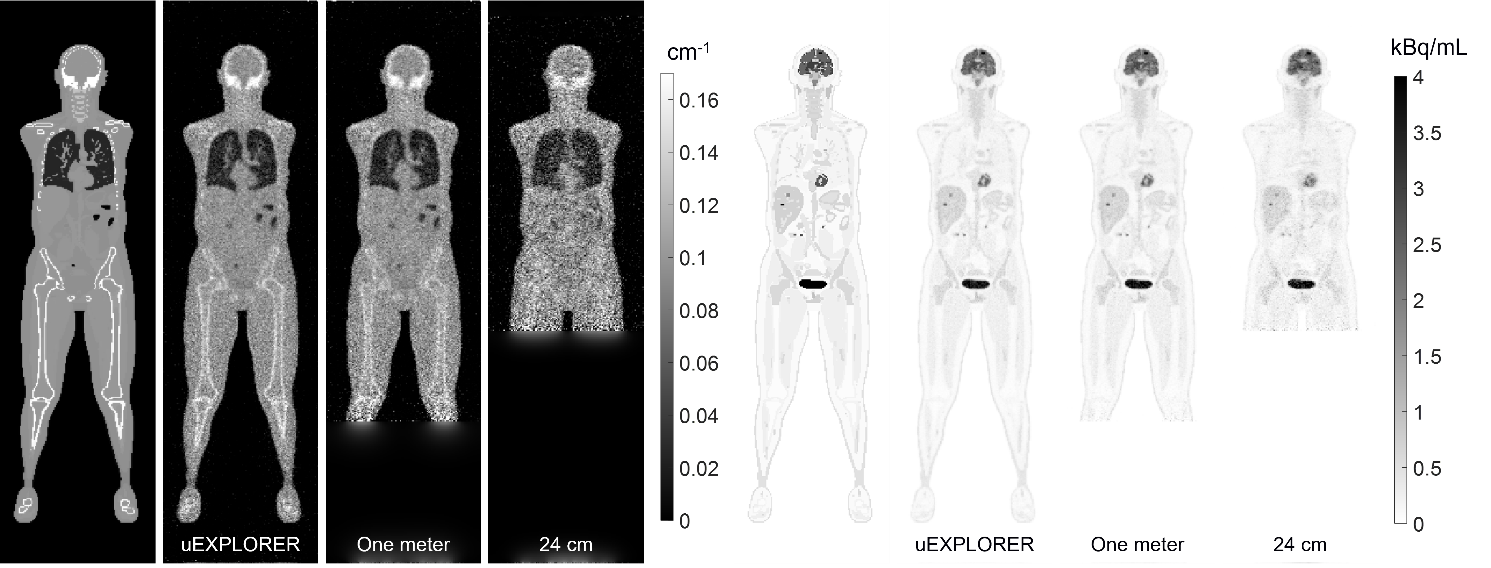

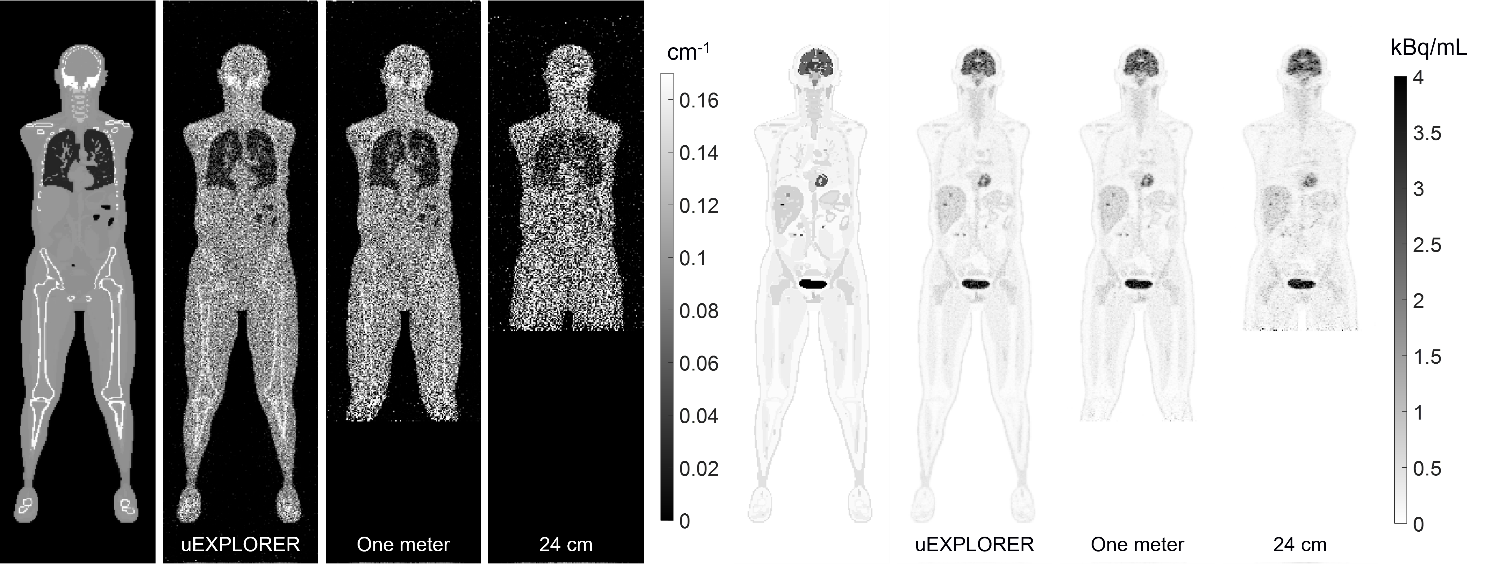


Supplementary Figure 11. Selected coronal slices for a 20-min whole-body scan on the three scanner geometries obtained from (top) MLTR reconstructions with no regularization, and OSEM reconstructions of the activity maps using MLTR-based μ-maps, (bottom) MLAA-TX reconstructions of activity and μ-maps with no regularization, initialized with regularized MLTR reconstructions.


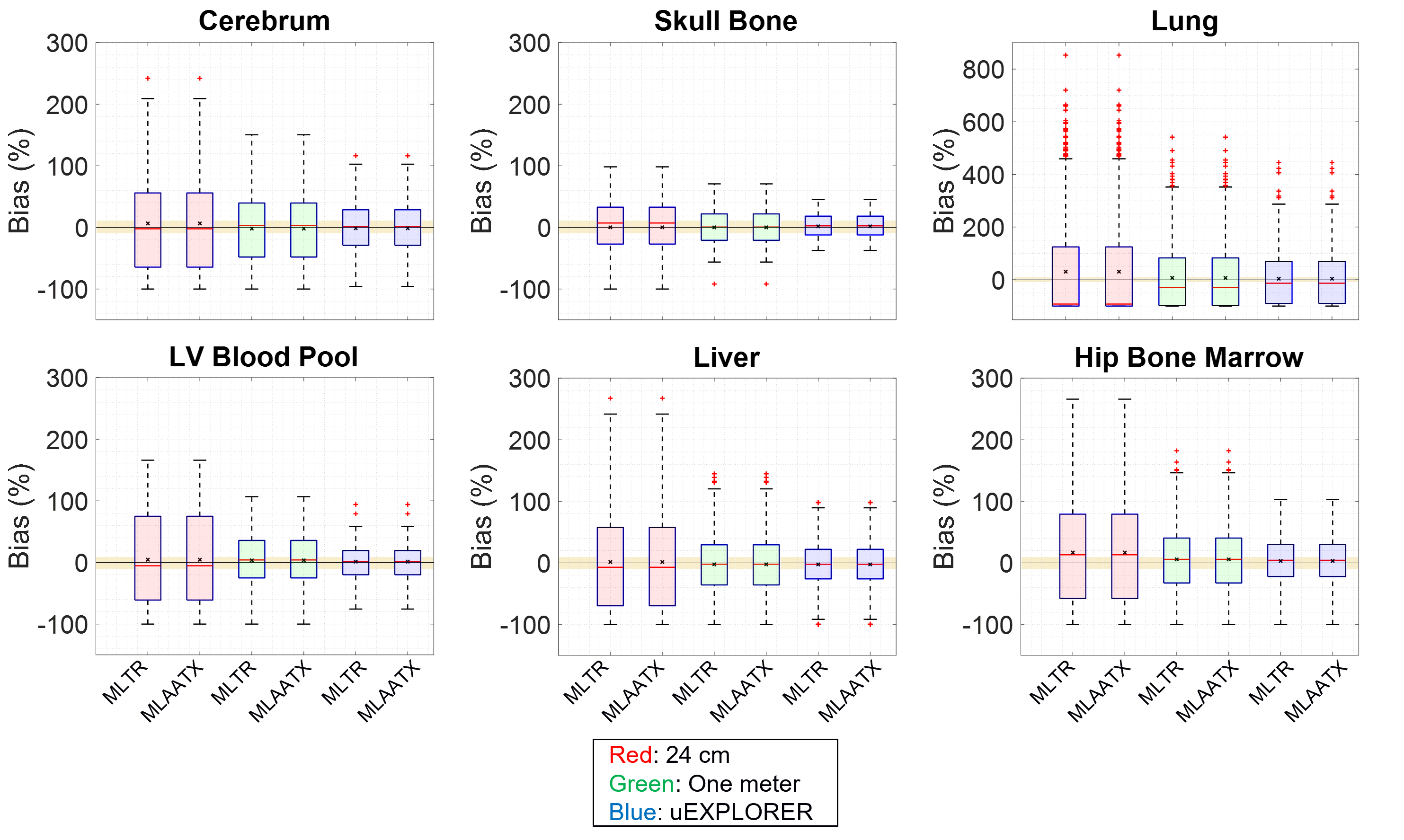


Supplementary Figure 12. Attenuation-map bias (%) distribution in selected organs of interest compared for the (red) 24-cm, (green) one-meter, and (blue) uEXPLORER scanners, using MLTR and MLAA-TX reconstructions with no regularization. The region marked with a yellow color depicts the ±10% range. Note: All box plots share the same labels. Results for the lungs are shown on a different y-axis range due to higher variability.


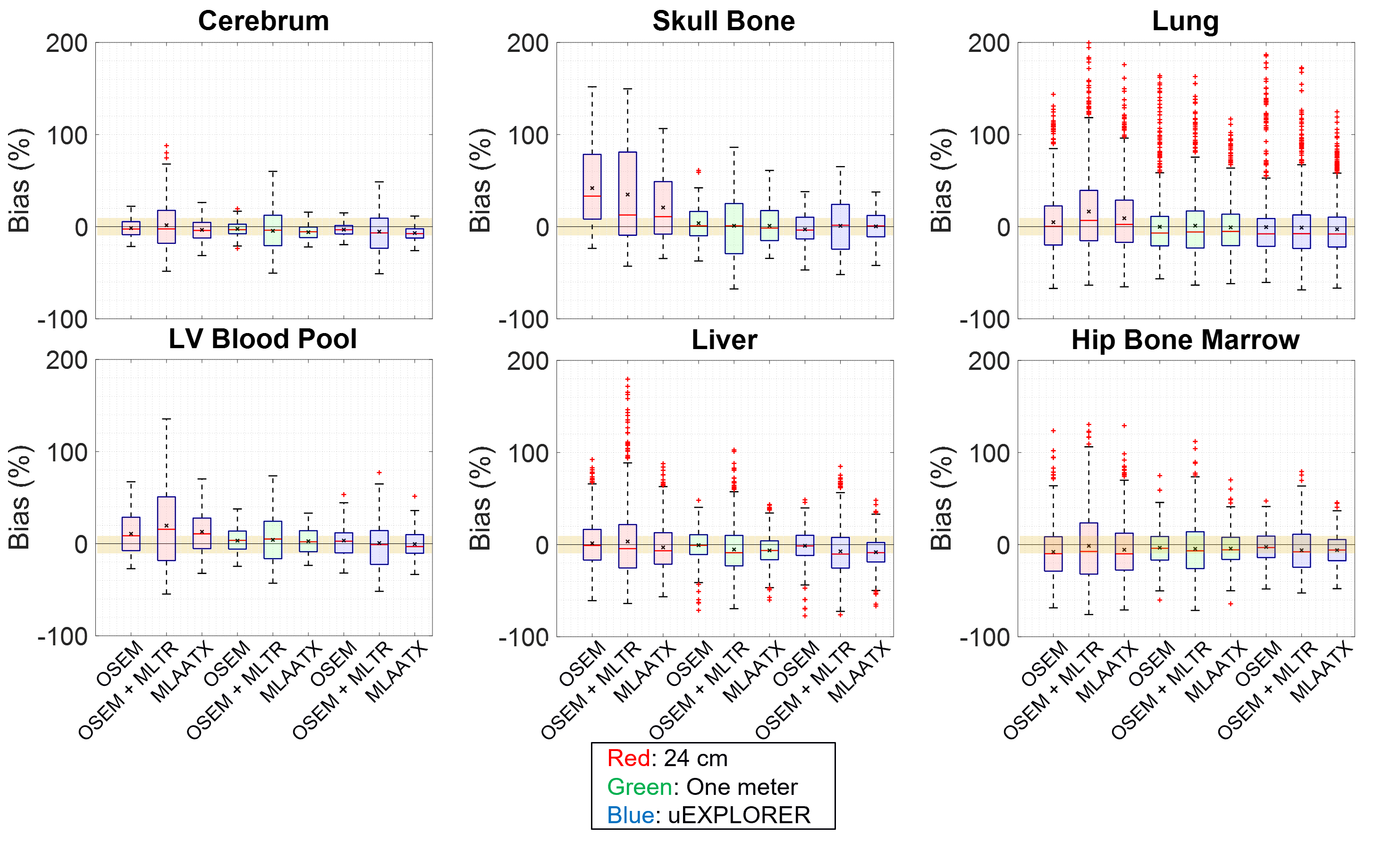


Supplementary Figure 13. Activity map bias (%) distribution in selected organs of interest compared for the (red) 24-cm, (green) one-meter, and (blue) uEXPLORER scanners, using the OSEM with the ground truth μ-map, OSEM with the MLTR-based μ-map with no regularization, and MLAATX with no regularization. The region marked with a yellow color depicts the ±10% range. Note: All box plots share the same labels.


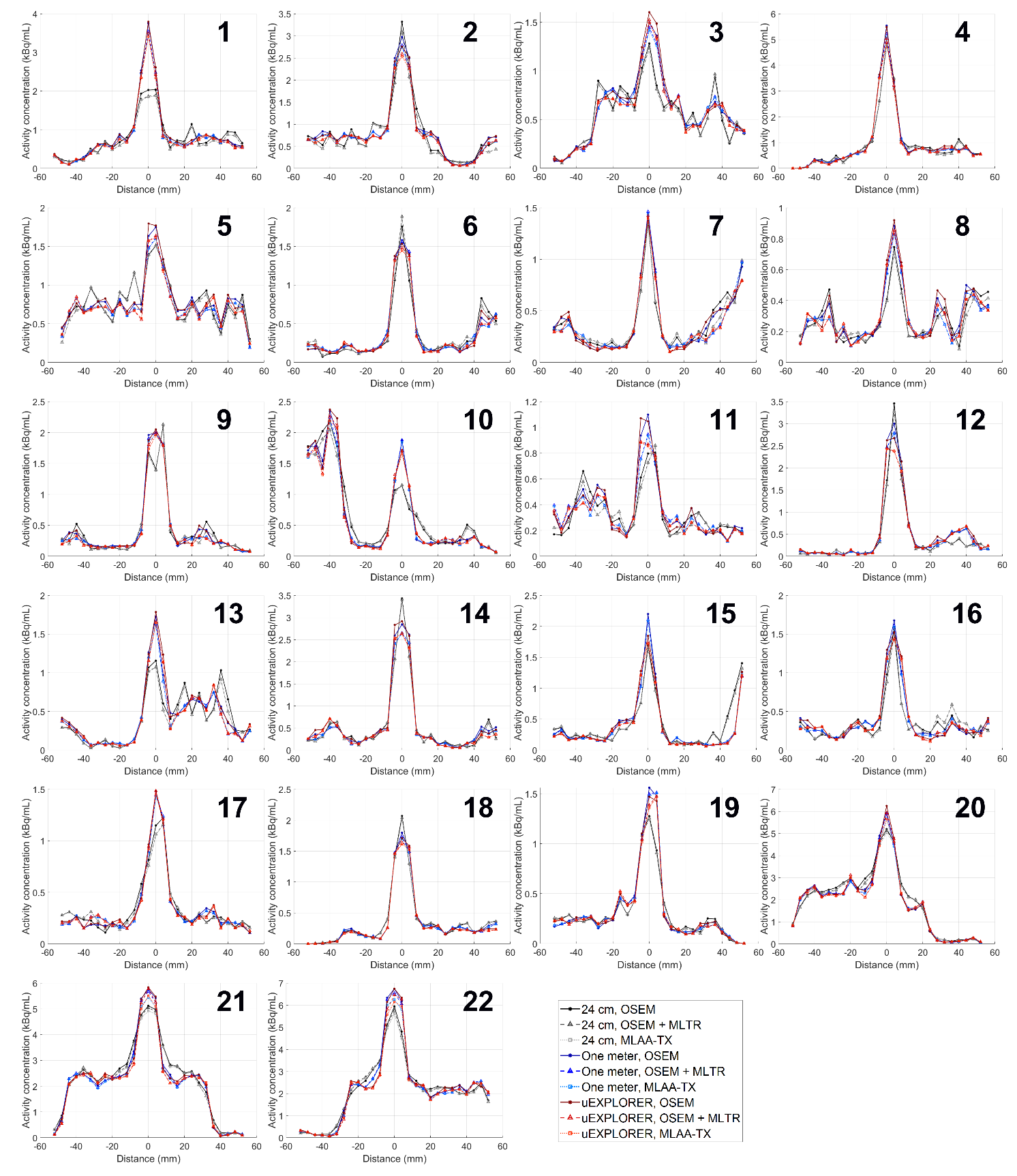


Supplementary Figure 14. 10-cm-long line profiles drawn horizontally on transverse slices through the center of the 22 lesions of the XCAT phantom, comparing OSEM with the ground truth μ-maps, OSEM with the regularized MLTR μ-maps, and regularized MLAA-TX, for the three scanner geometries.


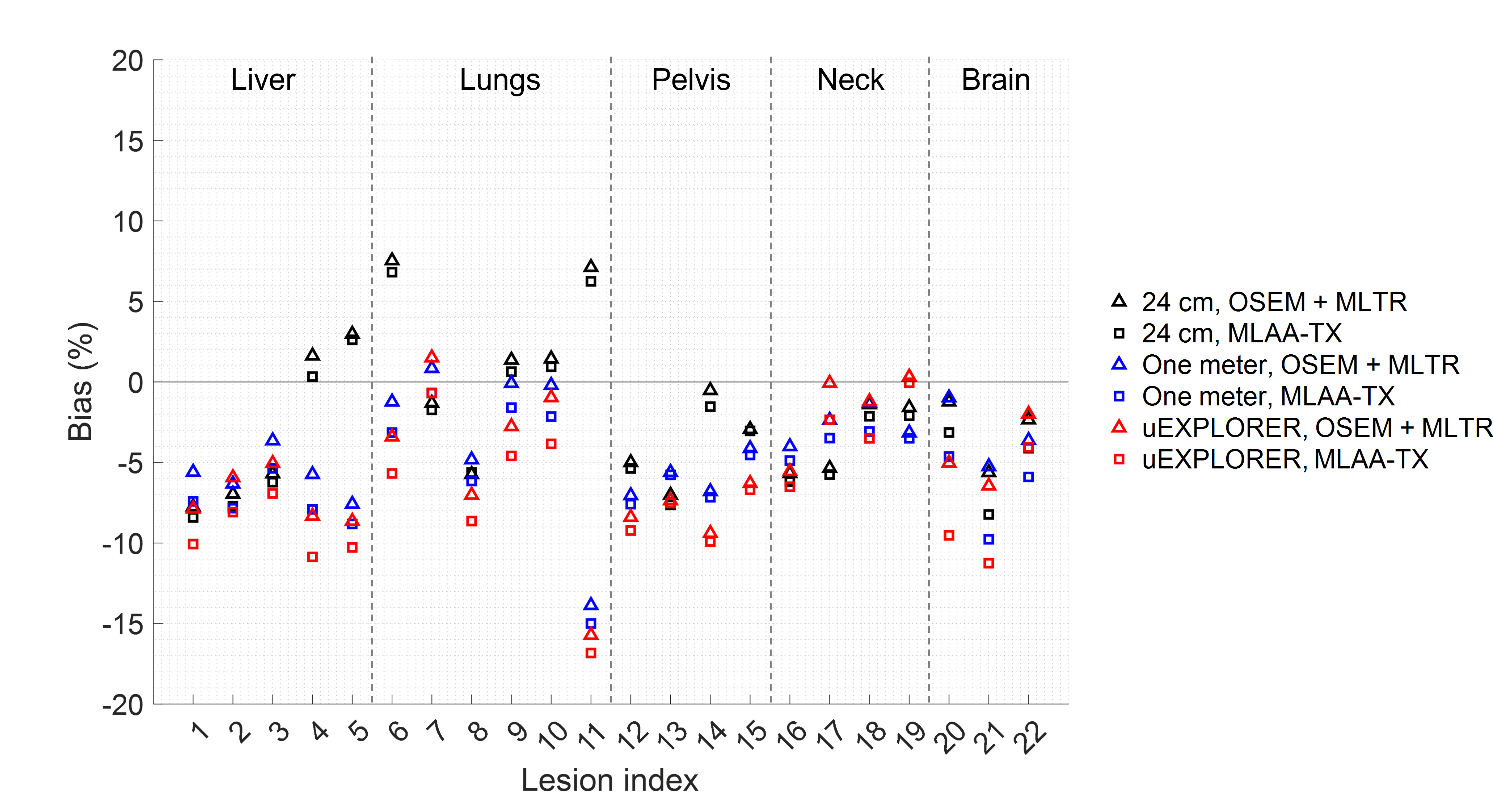


Supplementary Figure 15. Percentage bias of SUV_max_ in all 22 lesions of the XCAT phantom calculated in reference to the images reconstructed using the OSEM algorithm with the ground truth μ-maps, compared for the OSEM reconstructed images using the regularized MLTR μ-maps and regularized MLAA-TX reconstructions, shown for the three scanner geometries.


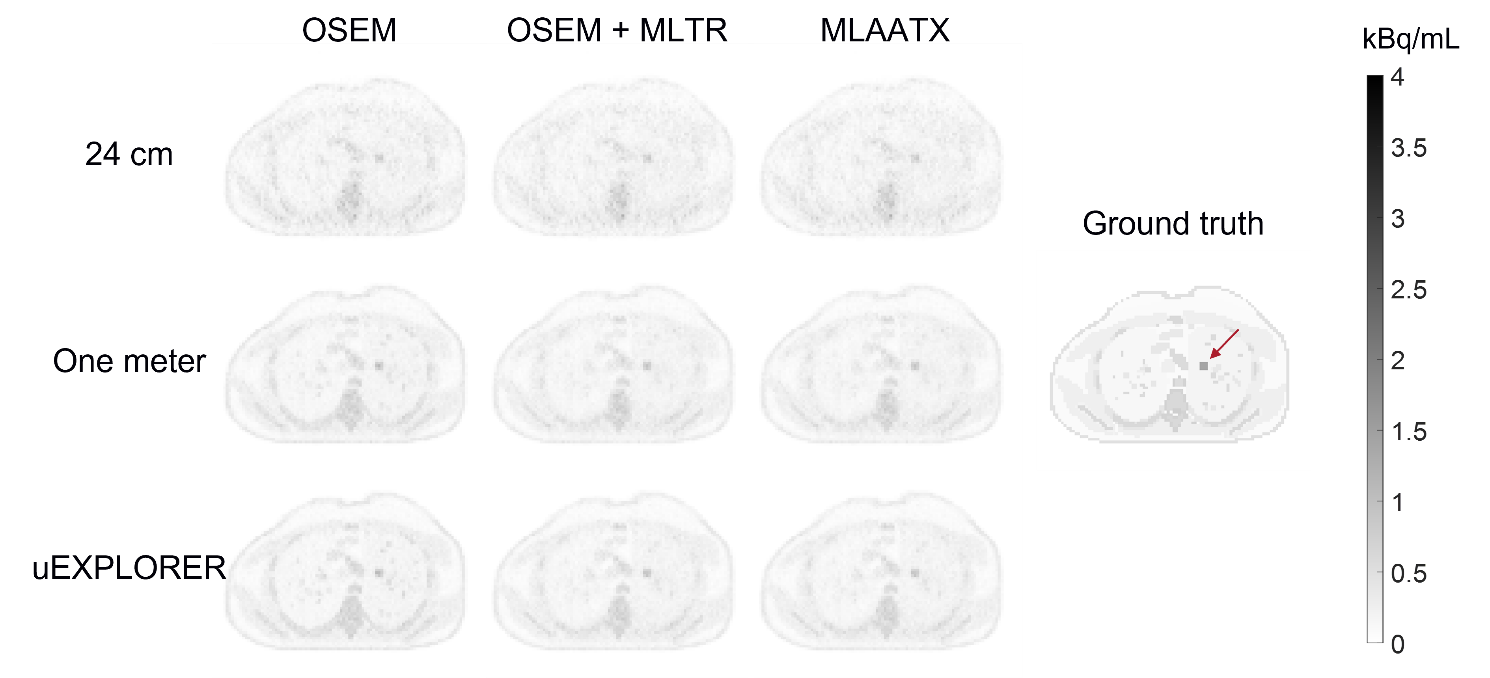


Supplementary Figure 16. Comparison of lesion contrast in the lung, showing an 8-mm lesion (lesion 11) in a transverse slice through lung reconstructed using OSEM with the ground truth μ-maps, OSEM with the regularized MLTR μ-maps, and regularized MLAA-TX, compared for the three scanner geometries and the ground truth activity map. The lesion location is marked with a red arrow on the ground truth image.


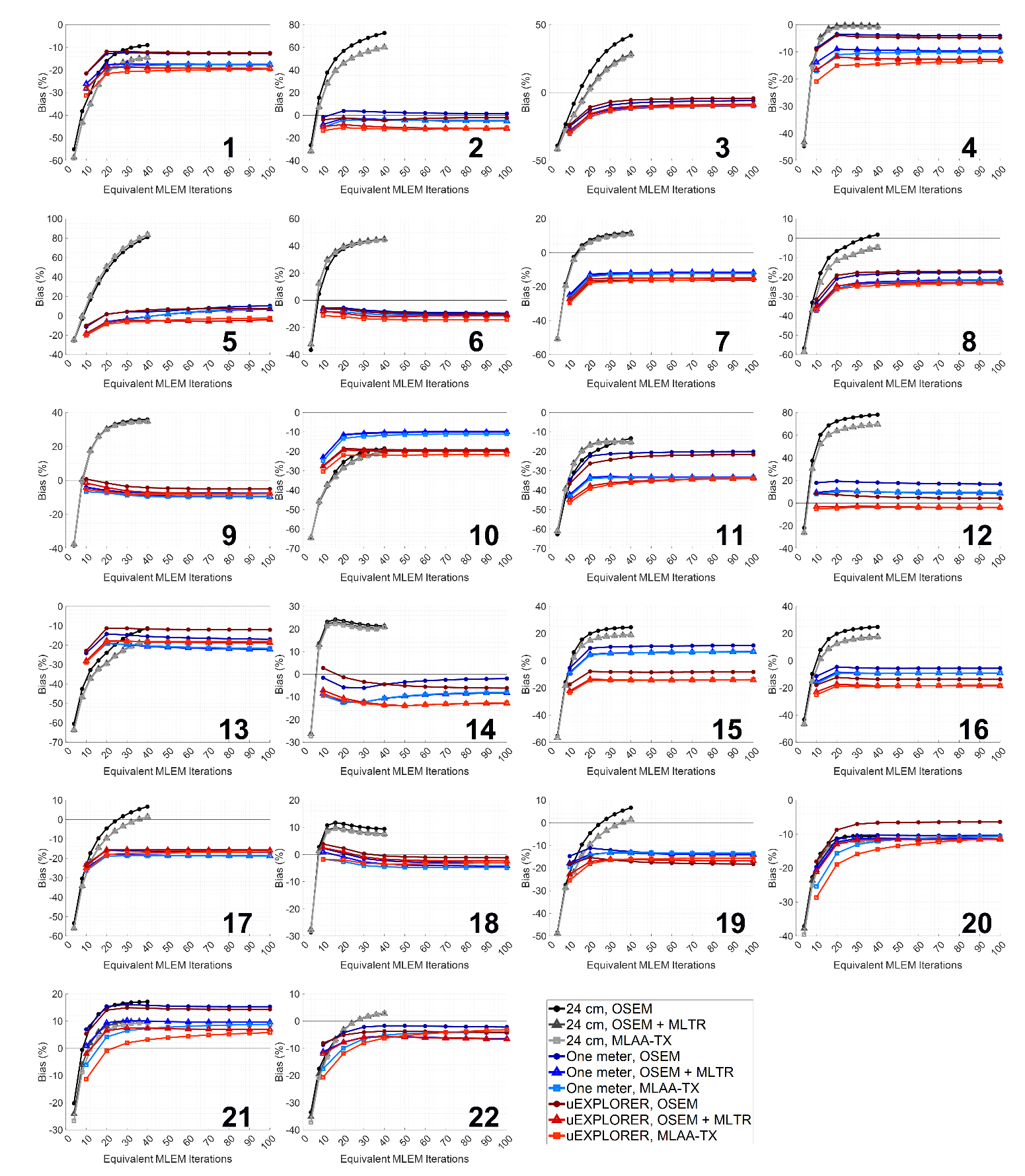


Supplementary Figure 17. Percentage bias of SUVmax in all 22 lesions of the XCAT phantom calculated in reference to the ground truth activity maps shown as a function of equivalent MLEM iterations, compared for the OSEM reconstructed images using the regularized MLTR μ-maps and regularized MLAA-TX reconstructions, for the three scanner geometries.

A

B

Energy of the second single (keV)

Energy of the second single (keV)

Supplementary Figure 18. Energy spectrum of the second single event detected in the lutetium 307 keV coincidence window shown separately for A) photons that did not go through Compton scattering before detection and B) photons that scattered before detection. The contribution from 511 keV photon emissions are not included.
